# Exploring *Erythrina* flavonoids as potential SARS-CoV-2 RdRp inhibitors through virtual screening, in silico ADMET evaluation, and molecular dynamics simulation studies

**DOI:** 10.1038/s41598-025-97311-w

**Published:** 2025-04-24

**Authors:** Tati Herlina, Vicki Nishinarizki, Abd. Wahid Rizaldi Akili, Ari Hardianto, Shabarni Gaffar, Muchtaridi Muchtaridi, Jalifah Latip

**Affiliations:** 1 Department of Chemistry, Faculty of Mathematics and Natural Sciences, Universitas Padjadjaran, Jatinangor, 45363 West Java Indonesia; 2 Faculty of Pharmacy, Universitas Padjadjaran, Jatinangor, 45363 West Java Indonesia; 3 Department of Chemical Sciences, Faculty of Science & Technology, Universiti Kebangsaan Malaysia (U.K.M.), 43600 Bangi, Selangor Malaysia

**Keywords:** Flavonoids, *Erythrina*, RNA-dependent RNA polymerase (RdRp), Structure-based bioinformatics, ADMET, Cheminformatics, Computational chemistry

## Abstract

The COVID-19 pandemic, caused by SARS-CoV-2, has intensified the search for effective antiviral agents. This study investigates the inhibitory potential of 473 flavonoids from the genus *Erythrina* against the key enzyme of SARS-CoV-2, RNA-dependent RNA polymerase (RdRp). Virtual screening campaign using molecular docking identified 128 flavonoids with stronger binding energies to RdRp than remdesivir, a WHO-endorsed drug. Lipinski’s Rule of Five and ADMET profiling suggested butein (**119**) as the promising RdRp inhibitor. Moreover, molecular dynamics simulations revealed that **119** binds effectively to RdRp and interacts with the RNA template and primer, suggesting a multi-faceted inhibitory mechanism. Our findings highlight the potential of Erythrina-derived flavonoids, particularly compound **119**, as potent RdRp inhibitors, warranting further experimental studies.

## Introduction

Severe Acute Respiratory Syndrome Coronavirus 2 (SARS-CoV-2) is the virus that causes the COVID-19 pandemic. It quickly infects vital human organs such as the lungs and heart^1–3^. Additionally, it may induce symptoms such as respiratory distress, which poses a significant risk and can be fatal^4^.

SARS-CoV-2 relies on RNA-dependent RNA polymerase (RdRp) complex to replicate its genome and transcribe its genes. RdRp comprises a main component called nsp12 and two additional subunits, nsp8 and nsp7^5^. It is the target of nucleoside-analogue-based inhibitors, such as ribavirin, remdesivir, and favipiravir^6–8^. Notably, the World Health Organization (WHO) endorses remdesivir, as studies have shown its efficacy in reducing mortality rates among non-ventilated COVID-19 patients^9^.

Several treatments derived from natural sources for SARS-CoV-2 are currently under investigation and development to ascertain their therapeutic efficacy^10–13^. One example is petatletin, a flavonoid isolated from *Tagetes patula*, which has the potential for anti-SARS-CoV-2 based on an in silico study^10^. Other in silico studies also suggest that flvononoid from *Selaginella tamariscina*, amentoflavone, can effectively inhibit RdRp^11,12^. In addition, an in vitro study showed that luteolin and quercetin can inhibit RdRp activity with IC_50_ of 4.6 ± 0.3 and 6.9 ± 1.0 µM respectively^14^. Therefore, flavonoids have great potential as anti-SARS-CoV-2 agents targeting the RdRp.

The genus *Erythrina*, spread in tropical and subtropical regions, is a potential source of flavonoids^15,16^. In 2009, Lee and colleagues showed that alpinumisoflavone, derived from the genus *Erythrina*, might hinder the growth of human immunodeficiency virus type 1^17^. Additionally, vitexin, another compound isolated from the genus *Erythrina*, has been suggested to suppress DNA expression in herpes simplex virus type 1^18^. However, the potential inhibitory effects of *Erythrina*-derived flavonoids on SARS-CoV-2 RdRp remain unexplored. Hence, an in silico study of flavonoids from the genus *Erythrina* to identify potential anti-SARS-CoV-2 agents is a promising area for investigation.

In this study, we screened 473 flavonoids isolated from the genus *Erythrina*^15,16^ against RdRp using molecular docking. Subsequently, we performed in silico absorption, distribution, metabolism, excretion, and toxicity (ADMET) to assess the pharmacokinetics and toxicities of the screened flavonoids. We also subjected the selected flavonoids to 250-ns molecular dynamics (MD) simulations to gain a deeper understanding of the molecular interaction aspect, unlocking their potency as SARS-CoV-2 RdRp inhibitors.

## Material and methods

### Preparation of flavonoid structures

The structures of the 473 flavonoids from the genus *Erythrina* were obtained from published literature, where these compounds have been thoroughly elucidated using various spectral techniques. The two-dimensional (2D) structures of the flavonoids were drawn using Chemaxon MarvinSketch, based on the literature^16^. The program was also used to predict the protonated states of these flavonoids at a physiological pH of 7.4. These three-dimensional (3D) structures, including their protonated states at physiological pH, were saved in the pdb file format. To prepare these 3D structures for molecular docking, MGLTools 1.5.6^19^ was employed.

### Molecular docking

The 3D structure of RdRp was obtained from the Protein Data Bank (PDB) website (https://www.rcsb.org), under the PDB ID 7BV2, as reported by Yin and coworkers^20^. Using BIOVIA Discovery Studio (DS) 2020 Client, the 3D structures of RdRp and its inhibitor, remdesivir monophosphate (F86), were extracted and saved as individual pdb files, while omitting water and ion molecules. These structures were then processed using AutoDockTools 1.5.6 to prepare them for molecular docking validation. Nonpolar hydrogen atoms were eliminated, while polar ones were kept. Gasteiger and Kollman atomic charges were applied to the F86 and RdRp structures, respectively. Active torsions for the F86 structure were configured as recommended by AutoDockTools 1.5.6. Both structures were then saved in pdbqt format. The grid box dimensions were defined to encompass the entire F86 structure. AutoGrid4.2 was used to calculate grid maps at RdRp’s binding site. For the molecular docking process, a Lamarckian genetic algorithm (GA) was chosen. The GA runs and population size were set at 100 and 300, respectively, while the maximum number of evaluations was capped at 2,500,000 (medium setting). All other search and docking parameters were left at their default settings. Molecular docking validation was carried out by redocking the F86 structure into RdRp’s binding site. The validation was deemed successful if the root mean square deviation (RMSD) for the docked F86 was less than 2.00 Å^15,21^.

The setup of flavonoid structures in AutoDockTools 1.5.6 was carried out in accordance with the procedures outlined for molecular docking validation. Virtual screening exercises were conducted using Raccoon v1.0b, operating on an Ubuntu 20.04 system. The grid map and docking parameters previously used for molecular docking validation served as the basis for these virtual screenings. The outcomes of the virtual screening were subsequently examined using AutoDockTools 1.5.6.

### Lipinski’s rule of five

The assessment of Lipinski’s Ro5 that was accessed on the SwissADME^22^ (http://www.swissadme.ch/) web server aims to evaluate whether hits from virtual screening campaigns meet the criteria of being an active drug orally. Several valuations are based on their molecular weights (MWs), logP values, and the number of hydrogen bond donors (HBDs) and acceptors (HBAs).

### ADMET prediction

ADMET (Absorption, Distribution, Metabolism, Excretion, and Toxicity) analysis was performed using the pkCSM web server (http://biosig.unimelb.edu.au/pkcsm)^23^. The absorption metrics included factors like water solubility, Caco2 permeability, human intestinal absorption, as well as P-glycoprotein substrate and inhibitors for P-glycoprotein I and II. For predicting the distribution of hits, parameters such as the steady-state volume of distribution (VDss), fraction unbound, blood–brain barrier (BBB), and central nervous system (CNS) permeabilities were considered. The computational metabolism assessment involved identifying hits as inhibitors for CYP1A2, CYP2C19, CYP2C9, and CYP2D6, along with their potential as substrates for CYP2D6 and CYP3A4. Criteria for evaluating excretion included total clearance and the role of renal organic cation transporter 2 (OCT2). To assess the safety profile, pkCSM was used to predict AMES toxicity and hepatotoxicity levels. The web server was also employed to estimate the efficacy of hits as hERG I and II inhibitors.

### Molecular dynamics simulations

The methodology for the MD simulation was adapted from our earlier works^24–26^. In summary, we determined the partial charges of secondary metabolite molecules using the Austin Model 1-Bond Charge Corrections (AM1-BCC) method, as implemented in the antechamber utility of AmberTools21^27^ (https://ambermd.org/AmberTools.php). Additional parameters for these secondary metabolites were sourced from the Generalized Amber Force Fields 2 (GAFF2). For all simulations, the ff19SB force field^28^ was applied to the amino acid residues of RdRp. Each RdRp-secondary metabolite complex was set up using the tleap utility in AmberTools21. During the preparation phase for MD, we employed the explicit SPC/E water model to solve each RdRp-ligand complex. A boundary box with dimensions of 10 Å was established. To achieve a physiological salt concentration of 0.15 M, a small number of Na^+^ and Cl﻿^–^ ions were added using the tleap utility in AmberTools21.

We utilized GPU-accelerated Particle-Mesh Ewald Molecular Dynamics (PMEMD) along with periodic boundary conditions, using the Amber20 software^27^, for each protein–ligand pairing. The process began with two consecutive energy minimization steps. During the first step, a restraint of 25 kcal mol^–1^ Å^–2^ was applied to the protein–ligand complex. This was followed by a second step where the restraint was reduced to 5 kcal mol^–1^ Å^–2^. The system temperature was then elevated to 300 K under a 50-ps NVT (Number-Volume-Temperature) condition. Subsequently, the simulation environment was switched to an NPT (Number-Pressure-Temperature) condition, and the system density was adjusted to 1 g cm^–3^ over a 50-ps period. In subsequent NVT simulations, the restraint on the solute was incrementally decreased by 1 kcal mol^–1^ Å^–2^ every 50 ps until it was completely removed.

For each system, we generated a 250-ns molecular dynamics (MD) trajectory by simulating at a temperature of 300 K under NPT (Number-Pressure–Temperature) conditions. The Particle-Mesh Ewald (PME) method was utilized to handle long-range electrostatic interactions, while a 10-Å cut-off was applied for short-range non-bonded interactions. All bonds that included hydrogen atoms were constrained using the SHAKE algorithm. The Langevin thermostat was employed to keep the temperature constant, and the Berendsen barostat was used for maintaining constant pressure in each system.

## Result and discussion

### Molecular docking

**Fig. 1:**
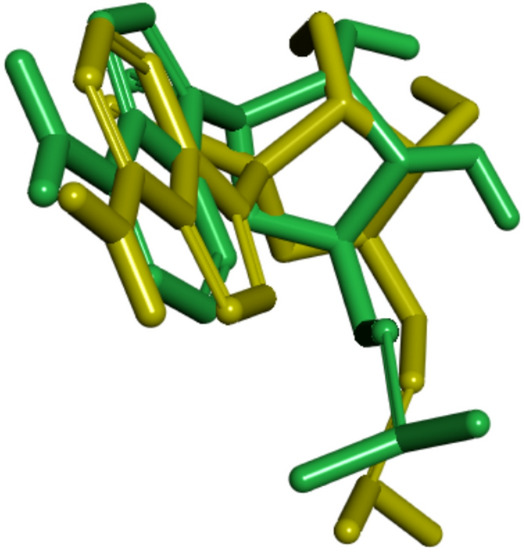
Visual comparison of the best remdesivir pose from molecular docking (green) with its 3D crystal structure pose (yellow).

Molecular docking validation was performed by redocking remdesivir to RdRp (PDB ID 7bv2) before conducting the virtual screening campaign. The grid box size used was 20 × 28 × 32 with a spacing of 0.375 Å to accommodate the binding active site pocket. The redocking procedure yielded the best remdesivir pose with an RMSD of 1.68 Å (Fig. 1), which satisfies the molecular docking validation criterion^15^. The best pose has a binding energy of –6.41 kcal mol^–1^.

Subsequently, 473 flavonoids isolated from the genus *Erythrina* (Table S1) were subjected to a virtual screening campaign against RdRp. The results show 128 flavonoids possessing binding energy values to RdRp stronger than remdesivir. These flavonoids include eight flavones, one flavonol, 29 flavanones, seven chalcones, eight iso-flavanes, thirteen isoflavanones, 31 isoflavones, 23 pterocarpans, two 6α-hydroxypterocarpans, one pterocarpan, two coumestans, and three 2-arylbenzofuranes.

Among all flavonoids, abyssinoside A (**379**) has the best binding energy score of –9.01 kcal/mol, followed by abyssinoside B (380) (–8.47 kcal mol^–1^), where both compounds are flavones. Abyssinone III (**32**), a flavanone, is at the third position of the hit list with a binding energy score of –8.13 kcal mol^–1^. The other 125 flavonoids possess binding energy scores between –6.41 and –8.00 kcal mol^–1^. As listed in Table 1, flavonones, isoflavanones, and pterocarpans are more promising RdRp inhibitors than other screened flavonoids since their binding energy scores are more negative than that of remdesivir.

**Table 1:** Seventeen top hits of flavonoids from the genus *Erythrina* against SARS-CoV-2 RdRp. The hits were screened from 473 flavonoids using molecular docking.

Structure	Group	Compound	Binding energy (kcal mol^–1^)
	Flavone	Abyssinoside A (**379**)	–9.01
	Flavone	Abyssinoside B (**380**)	–8.47
	Flavanone	Abyssinone III (**32**)	–8.13
	Isoflavone	Erythribyssin L (**325**)	–7.87
	Flavone	Vogelin C (**3**)	–7.74
	Chalcone	2,4,4ʹ-Trihydroxychalcone (**126**)	–7.67
	Ptereocarpan	Erybraedin D (**310**)	–7.64
	Ptereocarpan	Sophorapterocarpan A (Homoedudiol) (**290**)	–7.59
	Ptereocarpan	Erybraedin B (**308**)	–7.59
	Isoflavone	Ulexone A (**464**)	–7.59
	Chalcone	Isoliquiritigenin (**115**)	–7.57
	Ptereocarpan	Orientanol C (**319**)	–7.54
	Isoflavanone	Licoisoflavanone (**183**)	–7.52
	Ptereocarpan	Isoneorautenol (**321**)	–7.45
	Flavanone	Liquiritigenin-5ʹ-O-methyl ether (**26**)	–7.43
	Flavonol	3,7,4′-Trihydroxyflavone (**19**)	–7.42
	Chalcone	Butein (**119**)	–7.41

### Lipisnki’s rule of five (Ro5)

Next, we assessed the drug-likeness of the 128 hits according to Lipinski’s rule of five (Ro5) using the SwissADME webserver. The results suggest that 126 flavonoids satisfy all Ro5 criteria (Table S2): molecular weight less than 500 g mol, logP value less than five, and number of hydrogen bond acceptor and donor less than ten and five, respectively. Interestingly, **379** and **380**, the best hits (Table 1), violate three Ro5 criteria. Both compounds have molecular weights of 546.52 g mol^–1^ and hydrogen bond acceptor and donor numbers of twelve and eight, respectively. These results suggest that **379** and **380** may not be orally active drugs. Such an issue can be overcome using drug delivery systems like liposomes to increase solubility and bioavailability. The use of liposomes helps drug molecules to cross epithelial barriers, reaching target cells^29–31^.

### In silico absorption, distribution, metabolism, excretion, toxicity (ADMET)

Pharmacokinetics is an essential part of the development of oral drugs. Pharmacokinetics of drug candidates can be described by their absorption, distribution, metabolism, and excretion (ADME)^22^. Therefore, we predicted the pharmacokinetics of the 128 hits using the pkCSM web server^23^. Moreover, we also evaluated the toxicities of the hits through the same web server.

The absorption prediction results suggest that 128 hits can cross the human intestine (Table S3). However, another absorption parameter predicts that **379** and **380** may have low Caco2 permeabilities (Table S3). Both flavones may also have lower water solubility than 126 other flavonoids. Other absorption parameters are related to P-glycoprotein, which helps to predict drug bioavailability. The pkCSM web server suggests that 108 flavonoids are P-glycoprotein substrates, susceptible to being pumped back to the intestinal lumen, decreasing their absorption^32^. The web server also predicts that **63** and **62** flavonoids are P-glycoprotein I and II inhibitors, which increase absorption^32^. Interestingly, six of the 41 flavonoids predicted as P-glycoprotein I and II inhibitors are at the top 15 hits (Table 1). These flavonoids are **32**, erythribyssin L **(325**), erybraedin D (**310**), erybraedin B (**308**), ulexone A (**464**), and orientanol C (**319**).

The second pharmacokinetics parameter is the distribution, which is described by volume distribution steady state (VDss), fraction unbound in humans, central nervous system (CNS) permeability, and blood–brain barrier (BBB) permeability^15^. VDss is an important parameter that indicates whether drug molecules are more likely to remain in the plasma or to spread throughout the tissues. A drug with a high VDss value (log VDss > 0.45) is more likely to be distributed in the tissues rather than in the plasma, which may require higher drug doses. Conversely, if the VDss value is low (log VDss < –0.15), the drug is more likely to remain in the plasma^23^. The pkCSM web server predicts 17 flavonoids with low values of distribution volume (log VDss < –0.15) (Table  S4), including vogelin C (**3**) and isoliquiritigenin (**115**), which are at the top 17 hits (Table 1). Therefore, both hits may present more in plasma than tissue. Moreover, the other 14 hits have log VDss values between –0.64 and 0.388 (Table S4). One of them is **379**, the best hit, which may be moderately distributed between plasma and tissue (log VDss = 0.179). Nevertheless, all 128 hits have low unbound fraction values, reflecting their low bioavailability and inability to diffuse through cell membranes effectively^33^.

COVID-19 can influence the human brain^34^, since SARS-CoV-2 can infect the central nervous system (CNS)^35^. Interestingly, eight flavonoids at the top 17 hits are predicted to cross the CNS, indicated by logPS values of more than –2 and logBB values of more than –1 (Table S4). However, **379** and **380** may not readily penetrate the CNS since their logPS and logBB values are more than –3 and less than –1, respectively. Interestingly, carpachromene (**5**), which ranks 37th among the molecular docking hits (Table 1), is predicted to penetrate the blood–brain barrier easily.

Drug molecules can undergo metabolism before or after reaching their target cells. The process is crucial in activating or deactivating drug molecules. Furthermore, it is also vital for drug excretion from the body^36^. Cytochrome P450 enzymes, also known as the CYP. superfamily, play a significant role in drug metabolism, where CYP2C, CYP2D, and CYP3A subfamilies are the most active^15^. According to the pkCSM web-server prediction results (Table S5), the top 17 hits are not CYP2D6 substrate nor inhibitor. Five of them, sophorapterocarpan A (**290**), ulexone A (**464**), isoliquiritigenin (**115**), orientanol C (**319**), and isoneorautenol (**321**), are predicted as inhibitors for CYP1A2, CYP2C19, CYP2C9, and CYP3A4. Meanwhile, the pkCSM webserver predicts erythribyssin L (**325**), erybraedin D (**310**), and erybraedin B (**308**) as CYP2C19, CYP2C9, and CYP3A4 inhibitors. Therefore, they may inhibit substrate metabolism and clearance of the corresponding CYPs^23^. Interestingly, ulexone A (**464**), orientanol C (**319**), isoneorautenol (**321**), erythribyssin L (**325**), erybraedin D (**310**), and erybraedin B (**308**) are substrates for CYP3A4. These six flavonoids may also have low total clearances since their predicted logCLtot of less than 0.3 (Table S6).

Toxicity study is vital in drug discovery and development to minimize any side effects caused by drug molecules. All top 17 hits may not be toxic for the liver nor inhibitor for hERG I (Table S7). However, five of them are predicted to have AMES toxicities, suggesting their risk as mutagenic agents. Moreover, the pkCSM web server predicts other nine top hits as hERG II inhibitors, which may be dangerous for heart^37^. Interestingly, among the top 17 hits, only butein (**119**) is free from potential toxicity (TableS7). This prediction is further supported by an acute toxicity study of butein (**119**) in rats which reported that this compound is non-toxic with LD_50_ > 2000 mgkg﻿^–1^^38^. It is not a renal OCT substrate and has a low total clearance with logCLtot of 0.07, increasing its bioavailability. It is worth noting that **119** is predicted as a CYP1A2 inhibitor, which may play a role as a chemoprotective agent by reducing hepatoxicity mediated by the CYP1A2^39^. Meanwhile, **379**, the flavonoid with the strongest binding energy, may act as a mutagenic agent and cause cardiotoxicity.

### Molecular interactions between the selected hits and RdRp

**Fig. 2:**
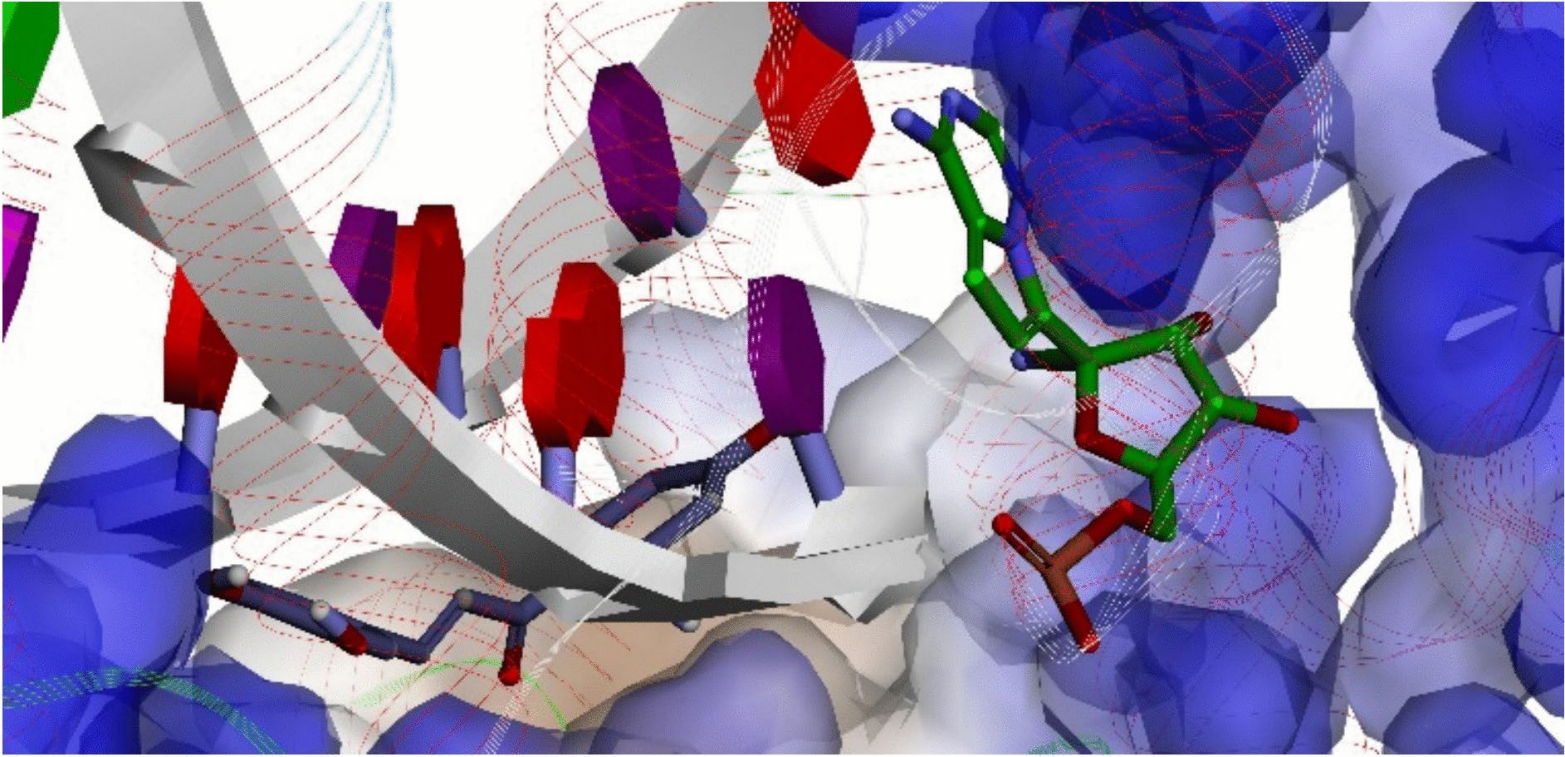
Different docking sites of **119** and remdesivir in the nsp12 of RdRp. Carbon atoms of **119** in grey licorice, whereas those of remdesivir are in green.

Subsequently, we examined the non-bonded interactions between compound **119** and the RdRp complex, focusing specifically on the nsp12 subunit. Intriguingly, our findings revealed that compound **119** uniquely binds the proteins. Unlike remdesivir, a well-known antiviral drug, compound **119** attaches to a completely different site on the nsp12 subunit, as illustrated in Fig. 2. Such a chalcone engages with both the RNA template and primer and interacts with specific amino acid residues in nsp12.

Furthermore, compound **119** demonstrates a multi-faceted interaction mechanism. It not only engages with specific amino acid residues in nsp12 but also forms interactions with the RNA template and primer. The interactions with the RNA template involve nucleotides U12, A13, and A14, while those with the RNA primer are mediated through nucleotides U18, U19, and U20, as detailed in Table S8. Hydrogen bonds (H-bonds) and π-π T-shaped hydrophobic interactions are the primary forces that stabilize the binding of compound **119** to the RNA template and primer. Meanwhile, with nsp12, **119** forms H-bonds with Gly590, Thr591, and Lys593. It also engages in hydrophobic interactions, specifically π-π T-shaped and π-alkyl interactions, with Ile589, Lys593, Ala688, and Leu758. The residues are located at the palm subdomain of RdRp. Similarly, **379** binds to the same palm subdomain of RdRp.

The palm subdomain is situated at the intersection of the fingers and thumb subdomain of RdRp. This particular subdomain accommodates the majority of the elements that are structurally preserved and participate in catalysis of RdRp^40^. The palm subdomain plays a crucial role in choosing NTP over deoxyribonucleoside triphosphate (dNTP). It also facilitates the phosphoryl transfer reaction by interacting with two metal ions, either magnesium (Mg^2+^) or manganese (Mn^2+^). Numerous groups of organic compounds have been developed to inhibit the palm domain of RdRp, including N-aryl uracil analogues, benzothiadiazines, acyl pyrrolidines, and benzofurans^41^. Notably, inhibitors binding to this subdomain have been previously observed in hepatitis C virus RdRp, such as 1-(2-cyclopropylethyl)-3-(1,1-dioxido-2h-1,2,4-benzothiadiazin-3-yl)-6-fluoro-4-hydroxy quinolin-2(1h)-one 30 and 5-cyclopropyl-2-(4-fluorophenyl)-6-[(2-hydroxyethyl)(methylsulfonyl)amino]-N-methyl-1-benzofuran-3-carboxamide^42^, with PDB IDs of 2GIK^43^ and 2BRK^44^, respectively. In terms of flavonoid, in silico study revealed that baicalein (a potential RdRp inhibitor) can bind to the palm subdomain of RdRp^45^.

### Molecular dynamics simulations

To better evaluate the binding of **119** to RdRp, we conducted a 250-ns molecular dynamics (MD) simulation. For comparative analysis, we also performed an MD simulation on baicalein which is a natural derived flavonoid which have been previously reported to inhibit the replication of SARS-CoV-2 with EC_50_ of 4.5 µM^45^. Additionally, MD simulations were carried out for ATP, the native substrate of RdRp, as well as for remdesivir in its triphosphate form (RTP).

### Root mean square deviation (RMSD)

**Fig. 3:**
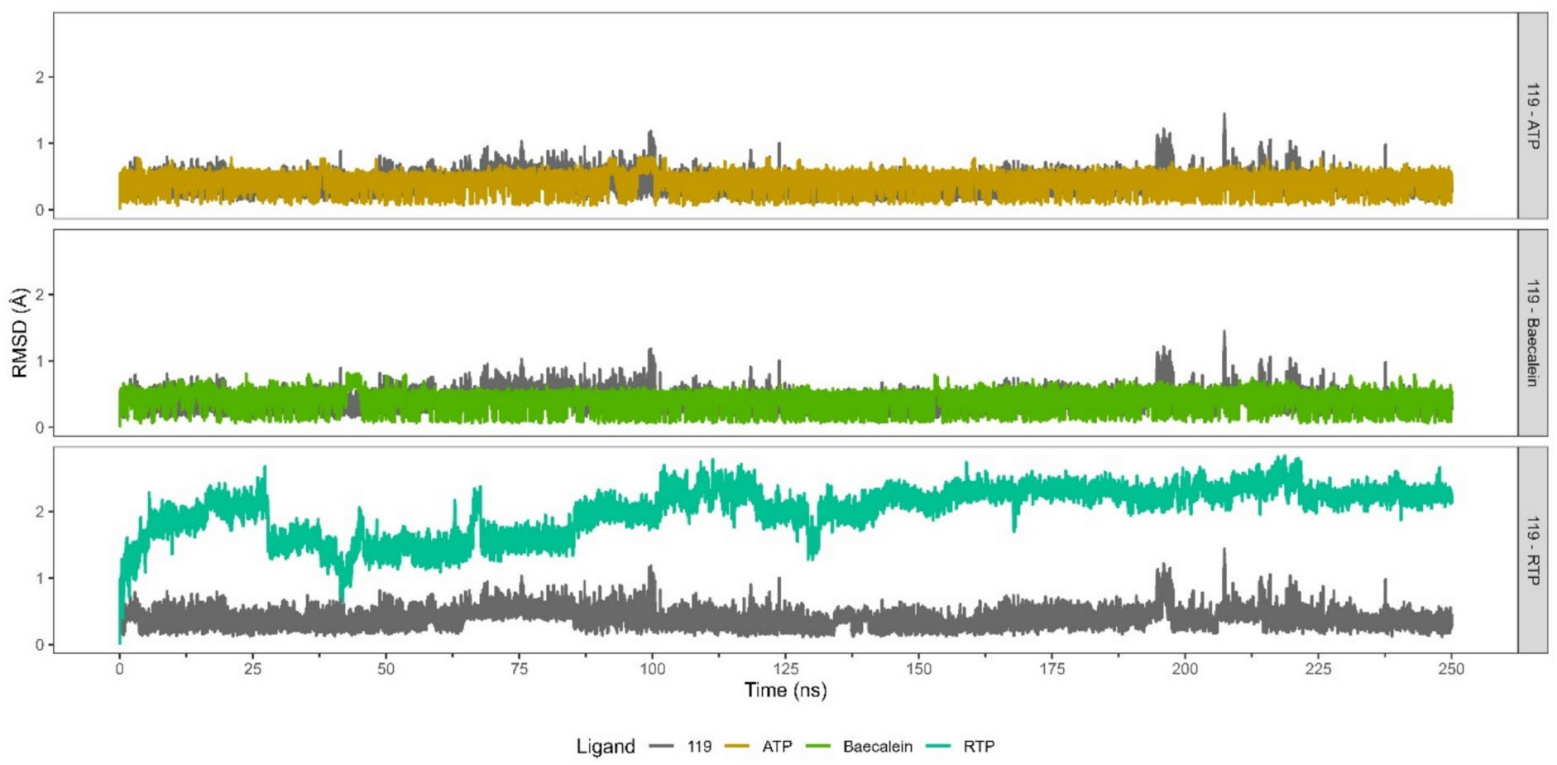
Comparative RMSD profiles of **119** and other ligands in complex with nsp12 of RdRp. Baicalein, ATP, and RTP were utilized for comparative analysis. The RMSD trajectories for ligand **119**, baicalein, ATP, and RTP are depicted by the grey, brown, green, and turquoise lines, respectively.

Stable Root Mean Square Deviation (RMSD) profiles serve as evidence for the consistent positioning of ligands within the receptor binding site, highlighting favorable ligand-receptor interactions^46,47^. Notably, **119** shows the lowest RMSD profile over ﻿baicalein, ATP, and RTP (Fig. 3). It has a median RMSD value of 0.375 Å (Table S9) and boasts the lowest median absolute deviation (MAD) value of 0.120 Å. These low values suggest that **119** maintains a stable conformation when bound to RdRp. This stability could potentially indicate favorable interactions between **119** and RdRp.

**Fig. 4:**
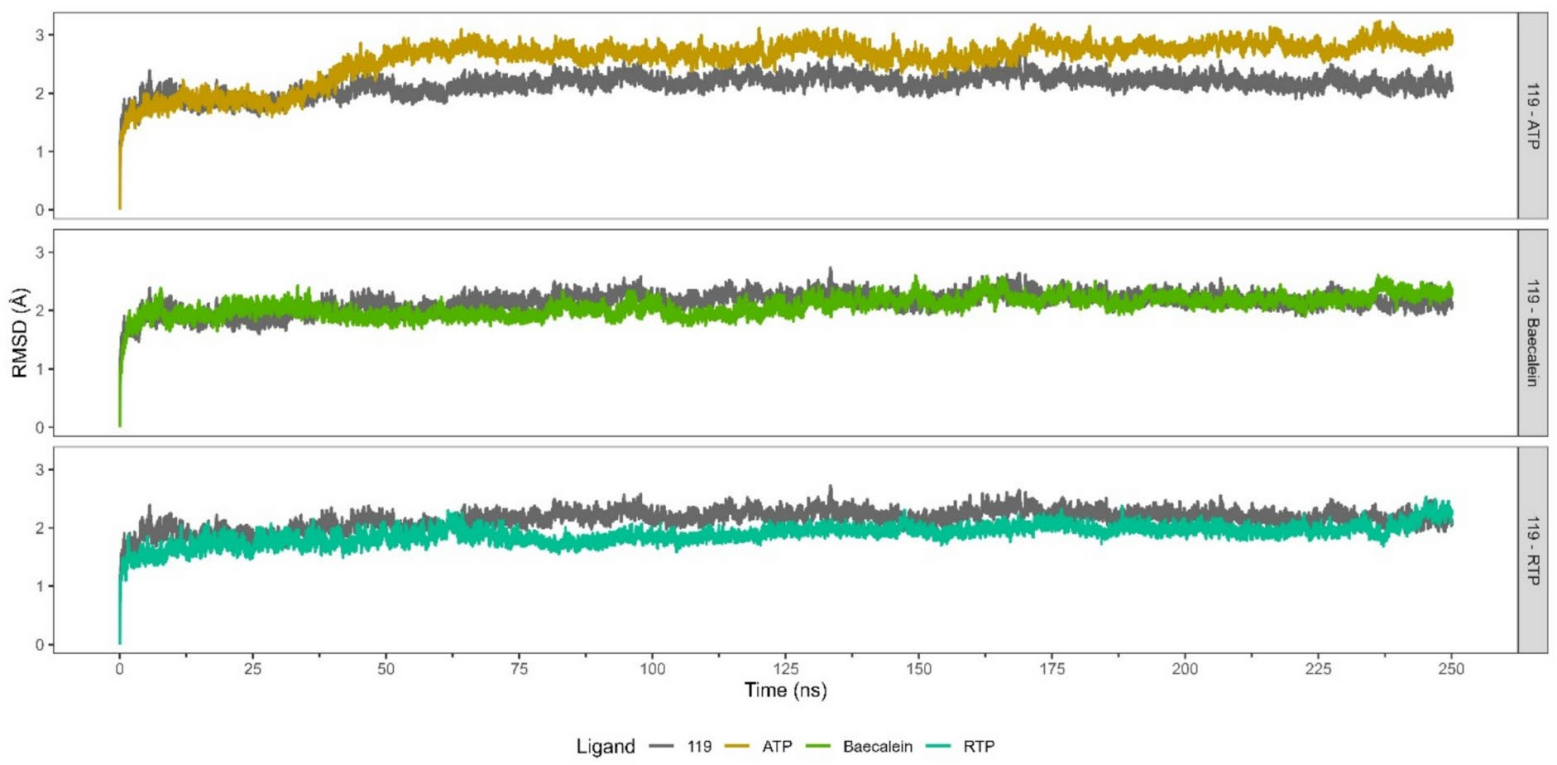
RMSD profiles and their clusters of RdRp (nsp12) binding **119** and other ligands as comparisons. The reference ligands are baicalein, ATP, and RTP.

From the perspective of the receptor (Table S10), RdRp bound to ATP displays higher RMSD values (median = 2.701 Å; MAD = 0.179 Å) compared to other complexes (Fig. 4). The complex of RdRp bound to baicalein and RTP display the lowest RMSD values of 2.083 ± 0.185 Å and 1.909 ± 0.138 Å respectively. The low RMSD value of the RdRp-baicalein complex supports the findings in the literature, which report that baicalein binds to RdRp which in turn inhibits its catalytic activity^45^. On the othe hand, RdRp bound to **119** displays slightly higher RMSD values of 2.171 ± 0.136 Å. The comparable stability of RdRp-**119** complex with that of RdRp-inhibitor complexes (RTP and baicalein) may suggest the potential inhibitory activity of **119** against RdRp.

Moreover, based on Hierarchical Cluster on Principal Component (HCPC) analysis, **119**-bound RdRp displays six conformation clusters, whereas both **379**- and ATP-bound RdRp complexes exhibit four clusters each. Additionally, the RTP-bound RdRp demonstrates a total of eight clusters. Although **119**-bound RdRp shows a higher number of conformation clusters than **379**- and ATP-bound RdRps, **119**-bound RdRp has a more consistent conformation cluster than the others. This consistent conformation cluster may correlate to favorable interactions between **119** and RdRp.

### Root mean square fluctuation (RMSF)

We subsequently carried out Root Mean Square Fluctuation (RMSF) analysis to delve deeper into dynamics of RdRp, particularly nsp12, after **119** binding. For comparative purposes, similar analyses were also performed on baicalein-, ATP-, and RTP-bound RdRp complexes. These analyses may provide information about protein–ligand interactions and the energetic information about the interactions^48^.

**Fig. 5:**
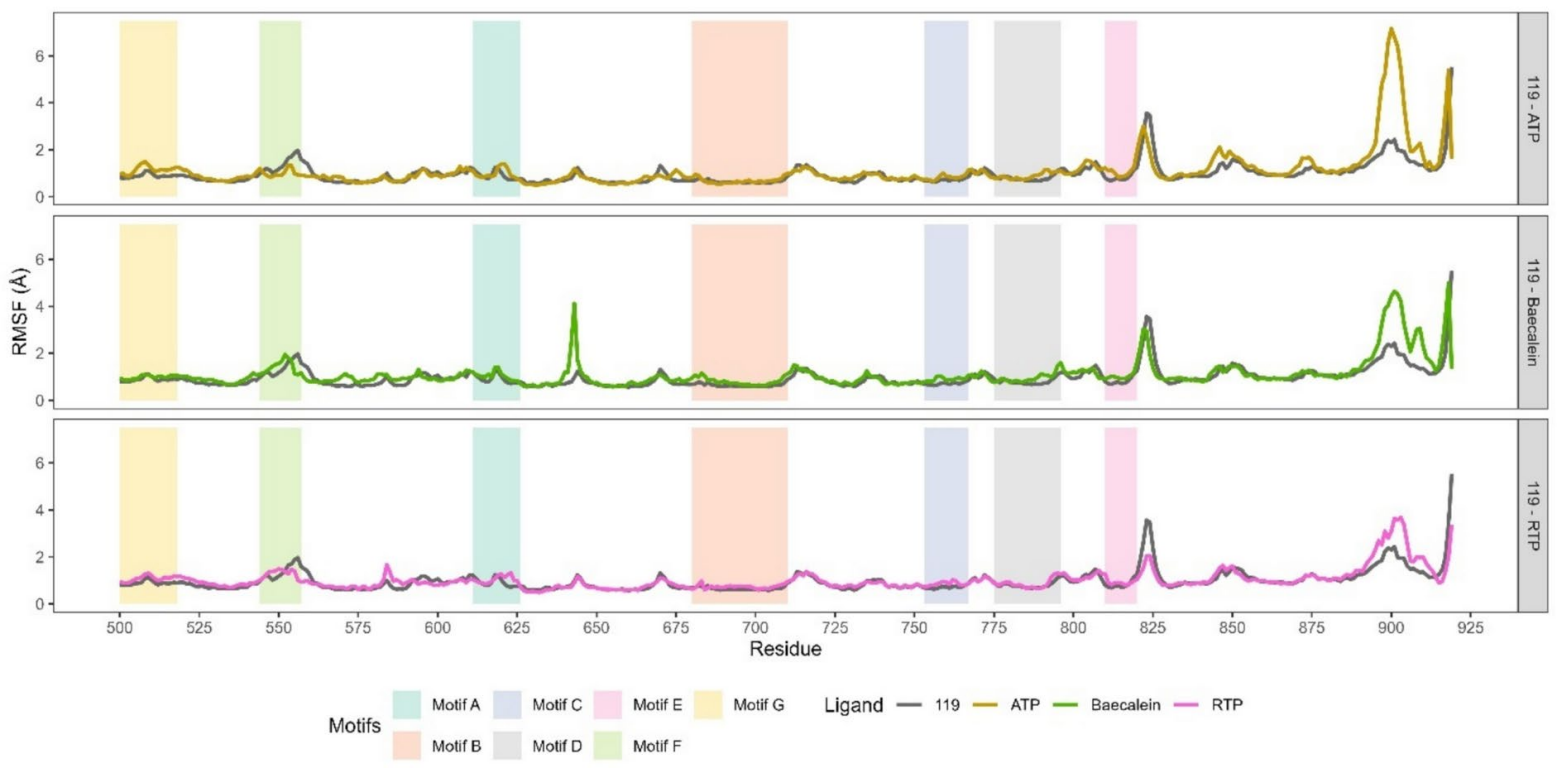
Pair‒wise RMSF profile comparation between RdRp binding **119** and other ligands. The RMSF profiles displayed belong the fingers, palm and thumb subdomains where motif A-G located.

The nsp12 subunit of RdRp features of seven distinct polymerase motifs, A-G (Fig. 5). These motifs are situated in the fingers, palm, and thumb subdomains. The RdRp complexes binding **119** and baicalein exhibit higher RMSF profiles at motif A (Thr611-M626) and the adjacent residues (Leu602-His613) compared to those bound to ATP and RTP. These elevated RMSF profile at the motif A, which houses the catalytic motif DX 2–4 D and is crucial for the catalytic activity of RdRp^20^, and the adjacent residues may be attributed to the absence of NTP in the RdRp-**119** and -baicalein MD systems.

For the rest motifs, RdRp binding **119** shows comparable RMSF profile to other RdRp complexes, with the exception of a small region in motif B, specifically between residues Tyr689 and Ile696. This flexible loop in motif B acts as a hinge and undergoes conformational changes related to template RNA and substrate binding. This motif is crucial for accommodating the RNA template during the replication process^49^. Interestingly, RdRp binding **119** exhibits higher RMSF values than those binding baecalein or ATP beyond motif E, around residue 850. A similar RMSF profile is also displayed by RdRp binding RTP. These variations in RMSF values may suggest unique binding interactions and dynamic behaviors at this specific ligand-binding site, which could have important implications for drug design or for understanding the mechanisms of RdRp inhibition.

### Molecular mechanic generalized surface area (MMGBSA) binding energy

**Fig. 6:**
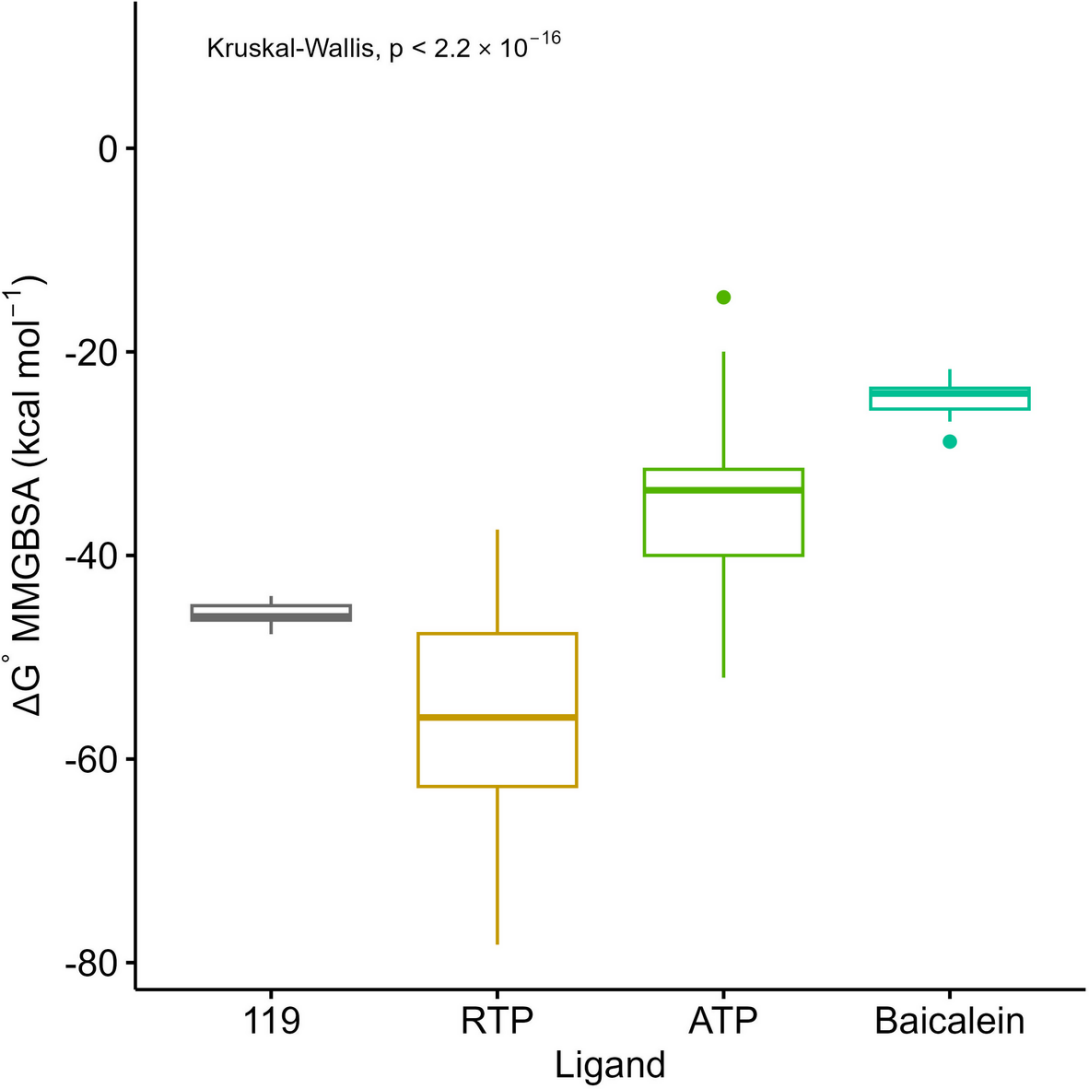
Box-plots of MMGBSA binding energy for ligands binding to RdRp. The Box-plots are from data points in Fig. S1. The Box-plots are complemented significant test results from a Dunn test, a post hoc of Kruskal–Wallis rank sum test.

The molecular mechanics-generalized Born surface area (MMGBSA) analysis of the RdRp binding ligands reveals significant differences in binding strength among these compounds. Notably, **119** exhibits a significant stronger binding affinity than ATP, the native ligand of RdRp. Additionally, **119** also exhibits lower binding energy compared to baicalein (p = 6.53 × 10^–4^, Dunn test, Fig. 6). This finding suggests that **119** may be a promising candidate for inhibiting the activity of RdRp, which is crucial for SARS-CoV-2 replication.

The strong MMGBSA binding energy of **119** (median = ‒45.98 kcal mol^–1^) is primarily derived from its van der Waals and electrostatic energies, which have median values of ‒50.97 and ‒18.95 kcal mol^–1^ (Table S11). Interestingly, the generalized born energy was positive at 29.21 kcal mol^–1^. This positive value serves to partially offset the negative contributions from the other energy components, highlighting the role of polar solvation in the overall binding process.

Comparing the potency of **119** to RTP, it is important to note that remdesivir is a known antiviral drug with demonstrated efficacy against SARS-CoV-2. The MMGBSA analysis suggests that RTP has higher binding affinity values than ligand **119**. This observation aligns with the clinical effectiveness of remdesivir, as it has been shown to inhibit the viral RNA polymerase and has been used as a treatment option for COVID-19 patients. While ligand **119** may exhibit strong binding affinity in silico, further experimental studies are necessary to determine its effectiveness in inhibiting SARS-CoV-2 RdRp and its potential as a therapeutic agent.

### MMGBSA per-residue energy decomposition and intermolecular interactions

**Fig. 7:**
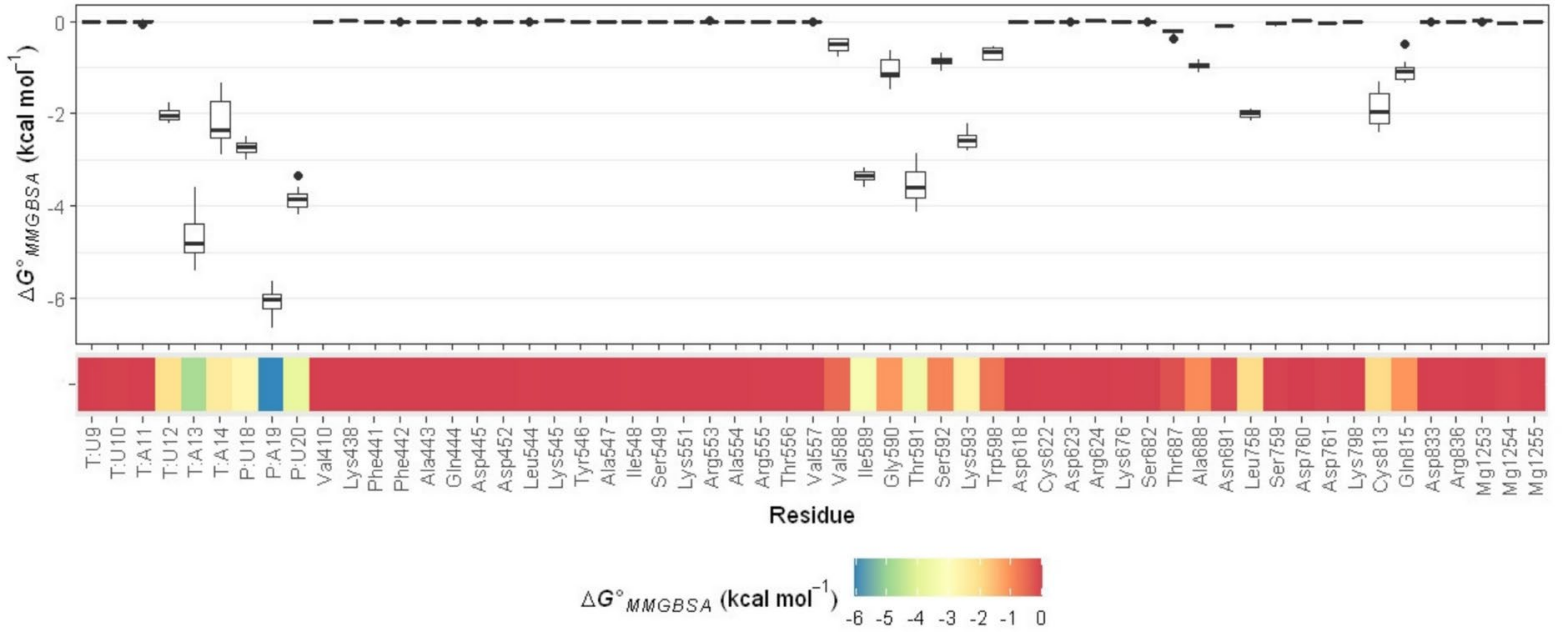
Box-plots and heatmap of MMGBSA binding energy decomposition for residues of RdRp and RNA template and primer interacting with **119**. T in nucleotide residues denotes the RNA template, whereas P is the RNA primer.

The analysis of MMGBSA per-residue energy decomposition aims to determine each residue contribution to the binding of **119** to RdRp. As shown in Fig. 7, the highest contribution to the binding of **119** originates from the RNA primer and template. The RNA primer nucleotide A19 and the template nucleotide A13 show the most favorable binding interactions with **119**, having median binding energy values of –6.02 and –4.80 kcal mol^–1^ (Table S12), respectively.

**Fig. 8:**
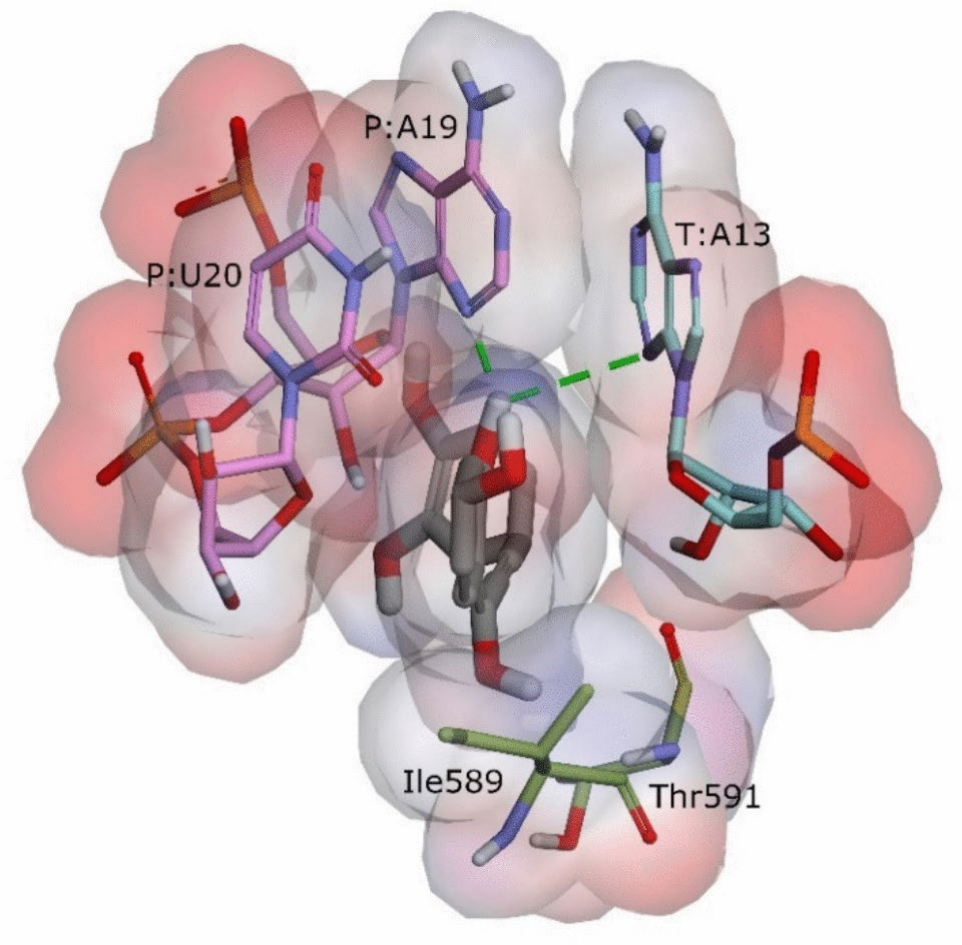
Interactions of **119** with RdRp and the RNA template and primer. Carbon atoms of RNA template and primer nucleotides are in cyan and pink, respectively, whereas those of amino acid residues of RdRp are in olive dark green. The green dash lines indicate H-bonds. Molecules surfaces of van der Waals are colored by interpolated charges.

Binding energy contributed by A19 mainly originates from electrostatics (–3.14 kcal mol^–1^) and var der Waals (–3.56 kcal mol^–1^) energy terms (Table S12). The electrostatics contribution is likely due to an H-bond between **119** and A19 (Fig. 8) with a conservation of 59.69% (Table S13). On the other hand, the substantial contribution from van der Waals energy can be attributed to the high degree of molecular surface complementarity between **119** and A19 (Fig. 8). Meanwhile, the interaction between **119** and A13 primarily is from van der Waals forces (–3.72 kcal mol^–1^) as shown in Table S12. Surprisingly, the electrostatics force causes unfavorable binding (0.32 kcal mol^–1^, Table S12) in the interaction between **119** and A13 through a transient H-bond with occurrence only 1.37% (Table S13). Furthermore, U20 from the RNA primer highly contributes to the binding energy of **119** by –3.85 kcal mol^–1^, where the highest portion is from van der Waals forces (–2.99 kcal mol^–1^, Table S12). These findings imply that interaction of **119** with A19 and U20 from the RNA primer and A13 from the RNA template may play a crucial role in the inhibition of RdRp.

Additional nucleotide residues also provide considerable binding energy contribution between –2.00 and –3.00 kcal mol^–1^ (Table S12). They are U18 (–2.73 kcal mol^–1^) from the RNA primer and A14 (–2.34 kcal mol^–1^) and U12 (–2.05 kcal mol^–1^) from the RNA template.

Although the per residue binding energy contributions from nsp12 are not as substantial as those from the RNA primer and template in the binding of **119**, the palm subdomain of nsp12 offers a greater number of contributing residues compared to the RNA nucleotides (Fig. 7). The most pronounced residues are Thr591 and Ile591, with binding energy contributions around –3.40 kcal mol^–1^ (Table S12). The binding stabilization formed by both residues mainly occurs from van der Waals forces as shown in Table S12

Overall, the stable binding of **119** or butein is due to its multifaced interactions with nsp12 residues and the RNA primer and template nucleotides. It infiltrates between the RNA primer and template while interacting with amino acid residues in the palm subdomain. Such binding mode interaction may halt RNA chain elongation, inhibiting SARS-CoV-2 RdRp activity.

## Conclusions

The study provides compelling evidence for the potential of *Erythrina*-derived flavonoids as inhibitors of the key enzyme of SARS-CoV-2, RNA-dependent RNA polymerase (RdRp). Out of 473 screened flavonoids using molecular docking approach, 128 exhibited stronger binding energies to RdRp than remdesivir. Butein (**119**) emerged as a particularly promising candidate, satisfying Lipinski’s Rule of Five and showing favorable ADMET profiles. Molecular dynamics simulations further substantiated the compound’s efficacy, revealing a multi-faceted inhibitory mechanism that involves interactions with both the RNA template and primer.

The study also delved into the pharmacokinetics and toxicity of the flavonoids, revealing that butein is free from potential toxicity and may have increased bioavailability. MMGBSA binding energy analysis indicated that butein has a stronger binding affinity to RdRp than ATP, the enzyme’s native substrate. The compound also demonstrated stable binding and favorable interactions, as evidenced by RMSD and RMSF analyses.

While the study is primarily computational, it lays a robust foundation for further experimental investigations. The findings are particularly timely and relevant, given the ongoing global health crisis caused by COVID-19. Butein could potentially serve as non-competitive inhibitor of RdRp. However, further in vitro studies are critical to confirm their inhibitory effects on SARS-CoV-2 RdRp and their impact on viral replication. Additionally, future research should explore the pharmacokinetics, pharmacodynamics, and safety profiles of butein to assess their viability in clinical settings.

## Supplementary Information

Supplementary Information.

## Data Availability

All data generated or analysed during this study are included in this published article (and its Supplementary Information files).
