# Supplementary material for: Exploring Erythrina flavonoids as potential SARS-CoV-2 RdRp inhibitors through virtual screening, in silico ADMET evaluation, and molecular dynamics simulation studies
Source: Sci Rep. 2025 Apr 24;15:14259. doi: 10.1038/s41598-025-97311-w (PMC12022342; doi:10.1038/s41598-025-97311-w)
Supplement: Supplementary file 1 — Supplementary Information. [file 41598_2025_97311_MOESM1_ESM.pdf]

Supplementary Materials for Article:

Exploring *Erythrina* Flavonoids as Potential SARS-CoV-2 RdRp Inhibitors through Virtual Screening, In Silico ADMET Evaluation, and Molecular Dynamics Simulation Studies

Tati Herlina [1,*], Abd. Wahid Rizaldi Akili [1], Vicki Nishinarizki [1], Ari Hardianto [1], Shabarni Gaffar [1], Muchtaridi Muchtaridi [2] and Jalifah Latip [3]

[1] Department of Chemistry, Faculty of Mathematics and Natural Sciences, Universitas Padjadjaran, Jatinangor 45363, West Java, Indonesia
[2] Faculty of Pharmacy, Universitas Padjadjaran, Jatinangor 45363, West Java, Indonesia
[3] Department of Chemical Sciences, Faculty of Science & Technology, Universiti Kebangsaan Malaysia (U.K.M.), Bangi 43600, Selangor, Malaysia
* Correspondence: tati.herlina@unpad.ac.id

Table S1. Molecular docking hits stronger than remdesivir monophospate. Molecular docking was performed by using AutoDock4.2 on 473 flavonoids from the genus *Eryhtrina*. 2
Table S2. Lipisnki's rule of five on 128 molecular docking hits. 9
Table S3. Absorption evaluation results of 128 molecular docking hits. 14
Table S4. Distribution evaluation results of 128 molecular docking hits. 20
Table S5. Metabolism evaluation results of 128 molecular docking hits. 25
Table S6. Excretion evaluation results of 128 molecular docking hits. 31
Table S7. Toxicity evaluation results of 128 molecular docking hits. 34
Table S8. Interactions between 119 and nsp12 of RdRp and the RNA template and primer. The prefix P indicates primer chain, while T denotes template chain. 38
Table S9. Descriptive statistics of RMSD values of RdRp-bound **119** and other reference ligands. The reference ligands are 379, adenosine triphosphaste (ATP), and remdesivir triphosphate (RTP). 39
Table S10. Descriptive statistics of RMSD values of RdRp (nsp12) binding butein **119** and other reference ligands. The reference ligands are **379**, adenosine triphosphaste (ATP), and remdesivir triphosphate (RTP). 39
Table S11. Median values of MMGBSA binding energy and their energy terms of 119, 379, ATP, and RTP to RdRp (nsp12). 39
Table S12. Median values of MMGBSA per-residue energy decomposition and their energy terms of **119** binding to RdRp (nsp12). 40
Table S13. H-bond occurrence formed between **119** and RdRp, the RNA primer and template. T in the Acceptor Residue column denotes the RNA template, whereas P is the RNA primer. 42

Table S1. Molecular docking hits stronger than remdesivir monophospate. Molecular docking was performed by using AutoDock4.2 on 473 flavonoids from the genus *Eryhtrina*.

	**Compound Name**		
**Group I**	**Flavones**	**Binding Energy (kcal/mol)**	**SMILES**
2	Luteolin	-6.69	OC1=CC2=C(C(O)=C1)C(=O)C=C(O2)C1=CC=C(O)C(O)=C1
3	Vogelin C	-7.74	C\C=C/CC1=C(O)C=C(O)C=C1C1=CC(=O)C2=C(O1)C(CC=C(C)C)=C(O)C=C2O
4	Vogelin J	-6.74	CC1(C)OC2=C(C=C1)C(O)=CC1=C2C(=O)C=C(O1)C1=CC=C(O)C=C1
5	Carpachromene	-7.04	COC1=CC=C(C=C1)C1=CC(=O)C2=C(OC)C3=C(OC(C)(C)C=C3)C=C2O1
379	Abyssinoside A	-9.01	[H]C1[C@@H](OC([H])(CO)[C@@]([H])(O)[C@]1([H])O)C1=C(O)C(=C2OC(=CC(=O)C2=C1O)C1=CC=C(O)C=C1)[C@]1([H])OC([H])(C)[C@@]([H])(O)C([H])(O)C1[H]
380	Abyssinoside B	-8.47	[H]C1[C@@H](OC([H])(CO)[C@@]([H])(O)C1([H])O)C1=C2OC(=CC(=O)C2=C(O)C(=C1O)[C@]1([H])OC([H])(C)[C@@]([H])(O)[C@]([H])(O)C1[H])C1=CC=C(O)C=C1
383	Apigenin	-7.14	OC1=CC=C(C=C1)C1=CC(=O)C2=C(O)C=C(O)C=C2O1
387	Vogeol	-6.75	CC(C)(C)C1CC2=C(O)C=C3OC(=CC(=O)C3=C2O1)C1=CC=C(O)C=C1
**Group II**	**Flavonol**	**Binding Energy (kcal/mol)**	**SMILES**
19	3,7,4′-Trihydroxyflavone	-7.42	OC1=CC=C(C=C1)C1=C(O)C(=O)C2=CC=C(O)C=C2O1
**Group III**	**Flavanones**	**Binding Energy (kcal/mol)**	**SMILES**
22	Liquiritigenin	-7.4	OC1=CC=C(C=C1)[C@@H]1CC(=O)C2=C(O1)C=C(O)C=C2
25	Erythribyssin K	-6.99	[H]C(=O)C1=C(O)C=CC(=C1)[C@@H]1CC(=O)C2=C(O1)C=C(O)C=C2
26	Liquiritigenin-5′-O-methyl ether	-7.43	COC1=C(O)C=CC(=C1)[C@@H]1CC(=O)C2=C(O1)C=C(O)C=C2
27	7,3′,4′-Trihydroxyflavanone	-7.4	OC1=CC2=C(C=C1)C(=O)C[C@H](O2)C1=CC=C(O)C(O)=C1
28	5′-(2-Hydroxy-3-methylbut-3-enyl) abyssinone II	-6.62	CC(C)=CCC1=CC(=CC(CC(O)C(C)=C)=C1O)[C@@H]1CC(=O)C2=C(O1)C=C(O)C=C2
30	Abyssinone I	-6.87	CC1(C)OC2=C(C=C1)C=C(C=C2)[C@@H]1CC(=O)C2=C(O1)C=C(O)C=C2
32	Abyssinone III	-8.13	CC(C)=CCC1=CC(=CC2=C1OC(C)(C)C=C2)[C@@H]1CC(=O)C2=C(O1)C=C(O)C=C2

35	Erylatissin C	-6.92	COC1=C(O)C=C(C=C1CC=C(C)C)[C@@H]1CC(=O)C2=C(O1)C=C(O)C=C2
37	7-Hydroxy-4′-methoxy-3′-(3-methylbut-2-enyl) flavanone	-6.44	COC1=CC=C(C=C1CC=C(C)C)[C@@H]1CC(=O)C2=C(O1)C=C(O)C=C2
41	Erythribyssin I	-7.4	CC1(C)OC2=CC=C(C=C2CC1O)[C@@H]1CC(=O)C2=C(O1)C=C(O)C=C2
43	Naringenin	-6.94	OC1=CC=C(C=C1)[C@@H]1CC(=O)C2=C(O1)C=C(O)C=C2O
45	Eriodictyol	-6.55	OC1=CC2=C(C(=O)C[C@H](O2)C2=CC=C(O)C(O)=C2)C(O)=C1
55	Burttinone	-6.93	COC1=C(CC=C(C)C)C=C(C=C1\C=C\C(C)(C)O)[C@@H]1CC(=O)C2=C(O1)C=C(O)C=C2O
57	Erycaffra F	-6.93	COC1=C(\C=C\C(C)(C)O)C=C(C=C1\C=C\C(C)(C)O)[C@@H]1CC(=O)C2=C(O1)C=C(O)C=C2O
68	2(S)-5,5′,7-Trihydroxy- [2″(5″-hydroxy)-methylpyrano]-(5″,6″:3′,4′) flavanone	-6.49	CC1(C)OC2=C(OO)C=C(C=C2C=C1)[C@@H]1CC(=O)C2=C(O1)C=C(O)C=C2O
69	2 (S)-5,7-Dihydroxy-3′-methoxy- [2″(5″-hydroxy)-methylpyrano]- (5″,6″:3′,4′) flavanone	-6.69	CCC1(C)OC2=C(OO)C=C(C=C2C=C1)[C@@H]1CC(=O)C2=C(O1)C=C(O)C=C2O
79	Fuscaflavanones B	-6.62	CC(C)=CCC1=C2OC3(C)COC(=C3)C2=C(O)C2=C1O[C@@H](CC2=O)C1=CC=C(O)C=C1
80	Abyssinin I	-6.55	COC1=CC(=CC2=C1OC(C)(C)C=C2)C1CC(=O)C2=C(O1)C=C(O)C=C2O
84	Abyssinoflavanone V	-7.12	CC1(C)OC2=C(C=C1)C=C(C=C2)C1CC(=O)C2=C(O1)C=C(O)C=C2O
85	Abyssinoflavanone VI	-6.79	CC(C)=CCC1=CC(=CC2=C1OCC2)C1CC(=O)C2=C(O1)C=C(O)C=C2O
90	2(S)-5,7-Dihydroxy- [2″,2″-(3″,4″-dihydroxy)-dimethylpyrano]-(5″,6″:3′,4′) flavanone	-6.47	CC1(C)OC2=CC=C(C=C2C(O)C1O)[C@@H]1CC(=O)C2=C(O1)C=C(O)C=C2O
92	2(S)-5,5′,7-Dihydroxy-6′-prenyl [2″,2″-(3″,4″-dihydroxy)-dimethylpyrano]-(5″,6″:3′,4′) flavanone	-7.1	CC(C)=CCC1=C(C=C2C(O)C(O)C(C)(C)OC2=C1O)[C@@H]1CC(=O)C2=C(O1)C=C(O)C=C2O
391	2S)-5,7-Dihydroxy-3′-prenyl-2″ξ-(4″-hydroxyisopropyl)dihydrofurano[1″,3″:4′,5′] flavanone	-6.45	CC(C)=CCC1=C2O[C@H](O)CC2=CC(=C1)[C@@H]1CC(=O)C2=C(O)C=C(O)C=C2O1
394	Erylatissin D	-6.46	CC(=C)C1CC2=C(O)C=C(C=C2O1)[C@@H]1CC(=O)C2=C(O)C=C(O)C=C2O1
396	Erylatissin G	-6.88	CC1(C)CCC2=C(O1)C(O)=CC(=C2)[C@@H]1CC(=O)C2=C(O)C=C(O)C=C2O1

398	2S-3’-(2-Hydroxy-3-methylbut-3-enyl)abyssinone II	-6.52	CC(C)=CCC1=C(O)C(CC(O)C(C)=C)=CC(=C1)[C@@H]1CC(=O)C2=CC=C(O)C=C2O1
401	4'-Hydroxy-6,3',5'-triprenylisoflavonone	-6.42	CC(C)=CCC1=CC(=CC(CC=C(C)C)=C1O)C1CC(=O)C2=C(O)C(CC=C(C)C)=C(O)C=C2O1
402	Mildbone	-6.79	CC1(C)CCC2=C3OC(CC(=O)C3=CC=C2O1)C1=CC=C(O)C(O)=C1
407	Sigmoidin L	-6.43	C[C@@H]1[C@H](C)C(C)(C)OC2=C1C=C(C=C2OC=C(C)C)C1CC(=O)C2=C(O)C=C(O)C=C2O1
**Group IV**	**Chalcones**	**Binding Energy (kcal/mol)**	**SMILES**
115	Isoliquiritigenin	-7.57	OC1=CC=C(\C=C\C(=O)C2=C(O)C=C(O)C=C2)C=C1
119	Butein	-7.41	OC1=CC(O)=C(C=C1)C(=O)\C=C\C1=CC=C(O)C(O)=C1
122	Abyssinone A	-6.88	CC1=CC(\C=C\C(=O)C2=C(O)C=C(O)C=C2)=CC2=C1OC(C)(C)C=C2
123	Abyssinone B	-6.5	CC1=CC(\C=C\C(=O)C2=C(O)C=C(O)C=C2)=CC2=C1OC(C)(C)C(O)C2
124	Abyssinone C	-6.76	CC1=CC(\C=C\C(=O)C2=C(O)C=C(O)C=C2)=CC2=C1OC(C)(C)C(O)C2O
126	2,4,4'-Trihydroxychalcone	-7.67	OC1=CC(O)=C(\C=C\C(=O)C2=C(O)C=C(O)C=C2)C=C1
416	Mildbone	-7.07	CC1(C)CCC2=C(O)C(=CC=C2O1)C(=O)\C=C\C1=CC=C(O)C(O)=C1
**Group V**	**Isoflavans**	**Binding Energy (kcal/mol)**	**SMILES**
130	Eryzerin D	-6.7	CC(C)=CCC1=C2OCC(CC2=CC2=C1OC(C)(C)C=C2)C1=CC=C(O)C=C1O
131	Eryzerin C	-7.03	CC1(C)OC2=C(C=C1)C=C1CC(COC1=C2)C1=CC=C(O)C=C1O
132	Eryvarin T	-7.19	COC1=CC(O)=C(OC)C=C1C1COC2=CC(O)=CC=C2C1
133	Erythribidin A	-7.31	CC1(C)OC2=C(C=C1)C(O)=CC=C2C1COC2=CC(O)=CC=C2C1
134	Phaseollinisoflavan	-7.01	CC1(C)OC2=CC=C(C3COC4=CC(O)=CC=C4C3)C(O)=C2C=C1
136	Erylivingstone J	-6.62	COC1=CC2=C(C=CC(C)(C)O2)C=C1C1COC2=CC(O)=CC=C2C1
137	Erylivingstone K	-6.53	CC1(C)OC2=C(C=C1)C=C(C1COC3=CC(O)=CC=C3C1)C(O)=C2
418	7,4'-Dihydroxy-2',5'-dimethoxyisoflavan	-6.81	COC1=CC(C2CCC3=CC=C(O)C=C3O2)=C(OC)C(O)=C1
**Group VII**	**Isoflavanones**	**Binding Energy (kcal/mol)**	**SMILES**
148	Eriotrichin B (Bidwillon A)	-6.56	CC(C)=CCC1=C(O)C(C)=C2OCC(C(=O)C2=C1)C1=CC=C(O)C=C1O

154	5,2',4'-Trihydroxy-6-prenyl-2′′′,2′′′dimethyldihydropyrano[5′′′,6′′′] isoflavanone	-6.44	COC1=C(CC=C(C)C)C=C2C(=O)C(COC2=C1C)C1=CC=C(O)C=C1O
155	5-Deoxyglasperin F	-7.23	CC1(C)OC2=C(C=C1)C(=CC=C2O)C1COC2=CC(O)=CC=C2C1=O
156	5-Deoxylicoisoflavanones	-7.1	CC1(C)OC2=CC=C(C3COC4=CC(O)=CC=C4C3=O)C(O)=C2C=C1
159	Sigmoidin J	-6.48	COC1=CC(O)=C(OC)C=C1C1COC2=CC(O)=C(CC=C(C)C)C=C2C1=O
160	Bidwillon B	-6.83	CC(C)=CCC1=C2OC(C)(C)C=CC2=CC2=C1OCC(C2=O)C1=CC=C(O)C=C1O
168	Eryzerin B	-6.5	CC(C)=CCC1=C2OCC(C(=O)C2=C2OC(C)(C)C(O)CC2=C1)C1=CC=C(O)C=C1
172	2,3-Dihydro-2′-hydroxyosajin	-6.57	CC(C)=CCC1=C2OC(C)(C)C=CC2=C2OCC(C(=O)C2=C1O)C1=CC=C(O)C=C1O
174	Erythraddison IV	-6.52	CC1(C)OC2=C(C=C1)C=C(C1COC3=CC(O)=CC(O)=C3C1=O)C(O)=C2
179	5,3′-Dihydroxy-2′′,2′′-dimethylpyrano-[5,6:6,7]-2′′′,2′′′-dimethylpyrano [5,6:5,4] isoflavanone	-6.98	CC1=CC(=CC2=C1OC(C)(C)C=C2)C1COC2=CC3=C(C=CC(C)(C)O3)C(O)=C2C1=O
181	5,4-Dihydroxy-2-methoxy-8-(3,3- dimethylallyl)-2,2-dimethylpyrano [5,6:6,7] isoflavanone	-6.45	COC1=CC(O)=CC=C1C1COC2=C(CC=C(C)C)C3=C(C=CC(C)(C)O3)C(O)=C2C1=O
183	Licoisoflavanones	-7.52	CC1(C)OC2=C(C=C1)C(O)=C(C=C2)C1COC2=CC(O)=CC(O)=C2C1=O
422	5,3’-Dihydroxy-5’-(3-hydroxy-3-methyl-1-butenyl)-4’-methoxy-2’’,2’’-dimethylpyrano [5,6:6,7] isoflavanone	-6.53	COC1=C(\C=C\C(C)(C)O)C=C(C=C1O)C1COC2=CC3=C(C=CC(C)(C)O3)C(O)=C2C1=O
**Group VIII**	**Isoflavones**	**Binding Energy (kcal/mol)**	**SMILES**
190	Daidzein	-6.43	OC1=CC=C(C=C1)C1=COC2=C(C=CC(O)=C2)C1=O
192	Neobavaisoflavone	-6.76	CC(C)=CCC1=C(O)C=CC(=C1)C1=COC2=C(C=CC(O)=C2)C1=O
195	Erythraddison II	-6.83	CC(C)=CCC1=CC2=C(OC=C(C2=O)C2=C(O)C=C(O)C=C2)C(CC=C(C)C)=C1O
196	2′,7-Dihydroxy-4′-methoxy-5′-(3- methylbut-2-enyl) isoflavone	-6.74	COC1=C(CC=C(C)C)C=C(C(O)=C1)C1=COC2=C(C=CC(O)=C2)C1=O

197	Calycosin	-6.69	COC1=C(O)C=C(C=C1)C1=COC2=C(C=CC(O)=C2)C1=O
200	Erylatissin B	-6.75	CC1(C)OC2=C(C=C1)C=C(C=C2O)C1=COC2=C(C=CC(O)=C2)C1=O
201	Corylin	-7.23	CC1(C)OC2=C(C=C1)C=C(C=C2)C1=COC2=C(C=CC(O)=C2)C1=O
202	Bidwillon C	-6.54	CC1(C)OC2=C(C=C1)C=C1C(=O)C(=COC1=C2)C1=CC=C(O)C=C1
209	Isowighteone (3′-Isoprenylgenistein)	-6.42	CC(C)=CCC1=C(O)C=CC(=C1)C1=COC2=C(C(O)=CC(O)=C2)C1=O
233	5,4'-Dimethoxy-3'-prenylbiochanin A	-6.78	COC1=CC(O)=CC2=C1C(=O)C(=CO2)C1=CC(O)=C(OC)C(\C=C\C(C)=C)=C1
234	Laburnetin	-6.58	CC(=C)C(O)CC1=C(O)C=C2OC=C(C3=CC=C(O)C=C3)C(=O)C2=C1
240	Indicanine E	-6.82	COC1=CC=C(C(O)=C1)C1=COC2=CC3=C(C=CC(C)(C)O3)C(OC)=C2C1=O
241	Parvisoflavone B	-6.48	CC1(C)OC2=C(C=C1)C(O)=C1C(=O)C(=COC1=C2)C1=CC=C(O)C=C1O
244	Auriculatin	-6.53	CC(C)=CCC1=C2OC=C(C(=O)C2=C(O)C2=C1OC(C)(C)C=C2)C1=CC=C(O)C=C1O
249	4′,7-Dihydroxy-2″,2″-dimethylpyrano [5″,6″:5,6] isoflavone	-6.63	CC1(C)OC2=C3C(=O)C(=COC3=CC(O)=C2C=C1)C1=CC=C(O)C=C1
252	Isoderrone	-6.53	CC1(C)OC2=C(C=C1)C=C(C=C2)C1=COC2=CC(O)=CC(O)=C2C1=O
253	Isochandalon	-6.9	CC(C)=CCC1=C(O)C=C2OC=C(C(=O)C2=C1O)C1=CC2=C(OC(C)(C)C=C2)C=C1
260	Erymildbraedin B	-6.58	C\C(C=O)=C\CC1=C2OC=C(C(=O)C2=C(O)C2=C1OC(C)(C)C=C2)C1=CC=C(O)C=C1
264	Erysenegalensein F	-6.64	CC1(C)OC1CC1=C2OC(C)(C)C=CC2=C(O)C2=C1OC=C(C2=O)C1=CC=C(O)C=C1O
267	Erysenegalensein L	-6.42	CC(=C)C(O)CC1=C2OC(C)(C)C=CC2=C(O)C2=C1OC=C(C2=O)C1=CC=C(O)C=C1O
271	5,7,4′-Trihydroxy-6-(2″-hydroxy3″-methylbut-3″enyl) isoflavone	-7.24	CC(=C)C(O)CC1=C(O)C2=C(OC=C(C2=O)C2=C(O)C=C(O)C=C2)C=C1O
287	4′-Hydroxyisoflavone-7-O-α-L-rhamnosyl/ (1 →6)-β-ᴅ-glucopyranoside	-6.75	OC1C(O)C(CO[*])OC(OC2=CC3=C(C=C2)C(=O)C(=CO3)C2=CC=C(O)C=C2)C1O
436	5,4'-Di-O-methylalpinumisofavone (Dimethylalpinumisoflavone)	-6.47	CCC1(C)OC2=C(C=C1)C(OC)=C1C(=O)C(=COC1=C2)C1=CC=C(OC)C=C1
440	Erydroogmansin B	-6.55	CC(C)=CCC1=C2OC=C(C(=O)C2=C(O)C=C1O)C1=CC=C2C=CC(C)(C)OC2=C1O
444	Erythraddison A	-6.68	CCC1(C)OC2=C(C=C1)C(O)=C1C(=O)C(=COC1=C2CO)C1=CC=C(O)C=C1
448	Erythrinin G	-6.68	CC1(C)OC2=C3C(=O)C(=COC3=CC(O)=C2C[C@H]1O)C1=CC=C(O)C=C1
451	Erythrivarone B	-6.9	CC1(C)CCC2=C3OC=C(C(=O)C3=C3OC(C)(C)CCC3=C2O1)C1=CC=C(O)C=C1

453	Eryvarin G	-6.58	CC(C)=CCC1=C2OC=C(OC3=C(O)C=C(O)C=C3)C(=O)C2=CC2=C1OC(C)(C)C=C2
454	Eryvarin X	-6.68	CC(C)=CCC1=C(O)C(CC=C(C)C)=C2OC=C(C(=O)C2=C1O)C1=CC=C(O)C=C1
458	3’-isoprenylgenistein	-6.54	CC(C)=CCC1=CC(=CC=C1O)C1=COC2=CC(O)=CC(O)=C2C1=O
464	Ulexone A	-7.59	CC(C)=CCC1=C2OC=C(C(=O)C2=CC=C1O)C1=CC=C2OC(C)(C)C=CC2=C1
**Group IX**	**Ptereocarpans**	**Binding Energy (kcal/mol)**	**SMILES**
289	Demethylmedicarpin	-6.9	[H][C@@]12COC3=C(C=CC(O)=C3)[C@]1([H])OC1=C2C=CC(O)=C1
290	Sophorapterocarpan A (Homoedudiol)	-7.59	[H][C@@]12COC3=C(C=CC(O)=C3)[C@]1([H])OC1=C2C=C(CC=C(C)C)C(O)=C1
293	Erythrabyssin II	-6.82	[H][C@@]12COC3=C(C=C(CC=C(C)C)C(O)=C3)[C@]1([H])OC1=C2C=CC(O)=C1CC=C(C)C
299	Erythribyssin B	-6.47	[H][C@@]12COC3=C(C=CC(O)=C3)[C@]1([H])OC1=C2C=CC(O)=C1C=O
300	Erythribyssin C	-6.75	[H][C@@]12COC3=C(C=C(CC=C(C)C)C(OC)=C3)[C@]1([H])OC1=C2C=C(OC)C(O)=C1
301	Medicarpin	-7.26	[H][C@@]12COC3=C(C=CC(O)=C3)[C@]1([H])OC1=C2C=CC(OC)=C1
308	Erybraedin B	-7.59	[H][C@@]12COC3=C(C=CC(O)=C3CC=C(C)C)[C@]1([H])OC1=C2C=CC2=C1C=CC(C)(C)O2
310	Erybraedin D	-7.64	[H][C@@]12COC3=C(C=CC(O)=C3CC=C(C)C)[C@]1([H])OC1=C2C=C2C=CC(C)(C)OC2=C1
312	Erybraedin F	-6.58	[H][C@@]12COC3=C(C=CC4=C3C=CC(C)(C)O4)[C@]1([H])OC1=C2C=C(OC)C(O)=C1
314	Erylysin A	-7.05	[H][C@@]12COC3=C(C=CC4=C3C=CC(C)(C)O4)[C@]1([H])OC1=C2C=CC2=C1CC(O)C(C)(C)O2
317	Shinpterocarpin	-6.45	[H][C@@]12COC3=C(C=CC4=C3C=CC(C)(C)O4)[C@]1([H])OC1=C2C=CC(O)=C1
319	Orientanol C	-7.54	[H][C@@]12COC3=C(C=C4C=CC(C)(C)OC4=C3)[C@]1([H])OC1=C2C=CC(O)=C1CC=C(C)C
320	Neorautenol	-6.83	[H][C@@]12COC3=C(C=C4C=CC(C)(C)OC4=C3)[C@]1([H])OC1=C2C=CC(O)=C1
321	Isoneorautenol	-7.45	[H][C@@]12COC3=C(C=CC(O)=C3)[C@]1([H])OC1=C2C=C2C=CC(C)(C)OC2=C1
322	8-Methoxyneorautenol	-6.64	[H][C@@]12COC3=C(C=C4C=CC(C)(C)OC4=C3)[C@]1([H])OC1=C2C=C(OC)C(O)=C1
323	Phaseollin	-7.08	[H][C@@]12COC3=C(C=CC(O)=C3)[C@]1([H])OC1=C2C=CC2=C1C=CC(C)(C)O2
324	Folitenol	-6.62	[H][C@@]12COC3=C(C=C(CC=C(C)C)C(O)=C3)[C@]1([H])OC1=C2C=CC2=C1C=CC(C)(C)O2
325	Erythribyssin L	-7.87	[H][C@@]12COC3=C(C=C4CC(O)C(C)(C)OC4=C3)[C@]1([H])OC1=C2C=CC(O)=C1CC=C(C)C
328	Erysubin C	-6.45	[H][C@@]12COC3=C(C=C(C=O)C(OC)=C3)[C@]1([H])OC1=C2C=CC(O)=C1
329	Erysubin D	-7.04	CC(C)=CCC1=CC2=C(OC[C@@]3([*])C4=C(O[C@@]23[*])C2=C(OC(C)(C)C(O)C2)C=C4)C=C1O
468	3,9-Dihydroxypterocarp-6a-ene	-7.36	OC1=CC2=C(C=C1)C1=C(O2)C2=CC=C(O)C=C2OC1
470	Erythrabissin II	-6.46	CC(C)=CCC1=C(O)C=C2OCC3C(OC4=C3C=CC(O)=C4CC=C(C)C)C2=C1
471	Gangetinin	-7.09	[H][C@@]12COC3=CC4=C(C=CC(C)(C)O4)C(OC)=C3[C@]1([H])OC1=C2C=CC2=C1C=CC(C)(C)O2

Group X	6α-Hydroxypterocarpans	Binding Energy (kcal/mol)	SMILES
333	Demethylerystagallin A	-6.79	CC(C)=CCC1=CC2=C(OC[C@]3(O)C2OC2=C3C=CC(O)=C2CC=C(C)C)C=C1O
341	Erysubin E	-6.76	CC(C)=CCC1=CC2=C(OC[C@]3(O)C2OC2=C3C=CC3=C2C=CC(C)(C)O3)C=C1O
**Group XI**	**Pterocarpene**	**Binding Energy (kcal/mol)**	**SMILES**
345	Erycristagallin	-6.47	CC(C)=CCC1=CC2=C(OCC3=C2OC2=C3C=CC(O)=C2CC=C(C)C)C=C1O
**Group XII**	**Coumestans**	**Binding Energy (kcal/mol)**	**SMILES**
352	Coumasterol	-7.35	OC1=CC2=C(C=C1)C1=C(O2)C2=C(OC1=O)C=C(O)C=C2
354	Sigmoidin K	-6.78	CC(C)=CCC1=CC2=C(OC(=O)C3=C2OC2=C3C=CC(O)=C2CC=C(C)C)C=C1O
**Group XV**	**2-Arylbenzofurans**	**Binding Energy (kcal/mol)**	**SMILES**
363	Vignafuran	-6.44	COC1=CC(O)=C(C)C=C1C1=CC2=C(O1)C=C(O)C=C2
368	Glyinflanin H	-6.47	CC1(C)OC2=C(C=C1)C(O)=C(C=C2)C1=CC2=C(O1)C=C(O)C=C2
372	2′-O-Demethylbidwillol B	-7.1	CC(C)=CCC1=C(O)C(=CC=C1O)C1=CC2=C(O1)C=C(O)C=C2

Table S2. Lipisnki's rule of five on 128 molecular docking hits.

Compound No	Compound	Molecular weight	Hydrogen bond acceptor	Hydrogen bond donor	Mlogp	Violation	Druglikeness
2	Luteolin	286.24	6	4	-0.03	0	Yes
3	Vogelin C	408.44	6	4	1.89	0	Yes
4	Vogelin J	335.34	5	2	1.64	0	Yes
5	Carpachromene	364.39	5	0	2.08	0	Yes
379	Abyssinoside A	546.52	12	8	-2.28	3	No
380	Abyssinoside B	546.52	12	8	-2.28	3	No
383	Apigenin	270.24	5	3	0.52	0	Yes
387	Vogeol	352.38	5	2	1.93	0	Yes
19	3,7,4′-Trihydroxyflavone	270.24	5	3	0.52	0	Yes
22	Liquiritigenin	256.25	4	2	1.27	0	Yes
25	Erythribyssin K	284.26	5	2	0.61	0	Yes
26	Liquiritigenin-5′-O-methyl ether	286.28	5	2	0.96	0	Yes
27	7,3′,4′-Trihydroxyflavanone	272.25	5	3	0.71	0	Yes
28	5′-(2-Hydroxy-3-methylbut-3-enyl) abyssinone II	422.51	6	5	2.76	0	Yes
30	Abyssinone I	322.35	4	1	2.38	0	Yes
32	Abyssinone III	390.47	4	1	3.38	0	Yes
35	Erylatissin C	354.4	5	2	2.04	0	Yes
37	7-Hydroxy-4′-methoxy-3′-(3-methylbut-2-enyl) flavanone	338.4	4	1	2.61	0	Yes
41	Erythribyssin I	340.37	5	2	1.63	0	Yes
43	Naringenin	272.25	5	3	0.71	0	Yes
45	Eriodictyol	288.25	6	4	0.16	0	Yes
55	Burttinone	438.51	6	3	2.21	0	Yes
57	Erycaffra F	454.51	7	4	1.41	0	Yes
68	2(S)-5,5′,7-Trihydroxy- [2″(5″- hydroxy)-methylpyrano]- (5″,6″:3′,4′) flavanone	370.35	7	3	1.29	0	Yes
69	2 (S)-5,7-Dihydroxy-3′-methoxy- [2″(5″-hydroxy)-methylpyrano]- (5″,6″:3′,4′) flavanone	384.38	7	3	1.51	0	Yes

Compound No	Compound	Molecular weight	Hydrogen bond acceptor	Hydrogen bond donor	Mlogp	Violation	Druglikeness
79	Fuscaflavanones B	420.45	6	2	2.01	0	Yes
80	Abyssinin I	368.38	6	2	1.5	0	Yes
84	Abyssinoflavanone V	338.35	5	2	1.82	0	Yes
85	Abyssinoflavanone VI	382.41	6	3	1.72	0	Yes
90	2(S)-5,7-Dihydroxy- [2″,2″-(3″,4″-dihydroxy)-dimethylpyrano]-(5″,6″:3′,4′) flavanone	372.37	7	4	0.29	0	Yes
92	2(S)-5,5′,7-Dihydroxy-6′-prenyl [2″,2″-(3″,4″-dihydroxy)-dimethylpyrano]-(5″,6″:3′,4′) flavanone	456.49	8	5	0.76	0	Yes
391	2S)-5,7-Dihydroxy-3′-prenyl-2″ξ-(4″-hydroxyisopropyl)dihydrofurano[1″,3″:4′,5′] flavanone	382.41	6	2	1.86	0	Yes
394	Erylatissin D	354.35	6	3	1.28	0	Yes
396	Erylatissin G	356.37	6	3	1.35	0	Yes
398	2S-3’-(2-Hydroxy-3-methylbut-3-enyl)abyssinone II	408.49	5	3	2.55	0	Yes
401	4'-Hydroxy-6,3',5'-triprenylisoflavonone	476.6	5	3	3.74	0	Yes
402	Mildbone	340.37	5	2	1.9	0	Yes
407	Sigmoidin L	438.51	6	2	2.58	0	Yes
115	Isoliquiritigenin	256.25	4	3	1.58	0	Yes
119	Butein	272.25	5	4	1.02	0	Yes
122	Abyssinone A	336.38	4	2	2.53	0	Yes
123	Abyssinone B	354.4	5	3	1.78	0	Yes
124	Abyssinone C	370.4	6	4	0.96	0	Yes
126	2,4,4'-Trihydroxychalcone	272.25	5	4	1.02	0	Yes
416	Mildbone	340.37	5	3	1.82	0	Yes
130	Eryzerin D	392.49	4	2	3.73	0	Yes
131	Eryzerin C	324.37	4	2	2.73	0	Yes
132	Eryvarin T	302.32	5	2	1.55	0	Yes
133	Erythribidin A	324.37	4	2	2.73	0	Yes
134	Phaseollinisoflavan	324.37	4	2	2.73	0	Yes
136	Erylivingstone J	338.4	4	1	2.95	0	Yes

Compound No	Compound	Molecular weight	Hydrogen bond acceptor	Hydrogen bond donor	Mlogp	Violation	Druglikeness
137	Erylivingstone K	324.37	4	2	2.73	0	Yes
418	7,4'-Dihydroxy-2',5'-dimethoxyisoflavan	302.32	5	2	1.55	0	Yes
148	Eriotrichin B (Bidwillon A)	354.4	5	3	2.04	0	Yes
154	5,2',4'-Trihydroxy-6-prenyl-2′′′,2′′′dimethyldihydropyrano[5′′′,6′′′] isoflavanone	368.42	5	2	2.26	0	Yes
155	5-Deoxyglasperin F	338.35	5	2	1.82	0	Yes
156	5-Deoxylicoisoflavanones	338.35	5	2	1.82	0	Yes
159	Sigmoidin J	384.42	6	2	1.72	0	Yes
160	Bidwillon B	406.47	5	2	2.82	0	Yes
168	Eryzerin B	408.49	5	2	2.63	0	Yes
172	2,3-Dihydro-2′-hydroxyosajin	422.47	6	3	2.28	0	Yes
174	Erythraddison IV	354.35	6	3	1.28	0	Yes
179	5,3′-Dihydroxy-2′′,2′′-dimethylpyrano-[5,6:6,7]-2′′′,2′′′-dimethylpyrano [5,6:5,4] isoflavanone	418.48	5	1	3.03	0	Yes
181	5,4-Dihydroxy-2-methoxy-8-(3,3- dimethylallyl)-2,2-dimethylpyrano [5,6:6,7] isoflavanone	436.5	6	2	2.48	0	Yes
183	Licoisoflavanones	354.35	6	3	1.28	0	Yes
422	5,3’-Dihydroxy-5’-(3-hydroxy-3-methyl-1-butenyl)-4’-methoxy-2’’,2’’- dimethylpyrano[5,6:6,7] isoflavanone	452.5	7	3	1.68	0	Yes
190	Daidzein	254.24	4	2	1.08	0	Yes
192	Neobavaisoflavone	322.35	4	2	2.2	0	Yes
195	Erythraddison II	406.47	5	3	2.64	0	Yes
196	2′,7-Dihydroxy-4′-methoxy-5′-(3- methylbut-2-enyl) isoflavone	352.38	5	2	1.86	0	Yes
197	Calycosin	284.26	5	2	0.77	0	Yes
200	Erylatissin B	336.34	5	2	1.64	0	Yes
201	Corylin	320.34	4	1	2.2	0	Yes
202	Bidwillon C	320.34	4	1	2.2	0	Yes
209	Isowighteone (3′-Isoprenylgenistein)	338.35	5	3	1.64	0	Yes

Compound No	Compound	Molecular weight	Hydrogen bond acceptor	Hydrogen bond donor	Mlogp	Violation	Druglikeness
233	5,4'-Dimethoxy-3'-prenylbiochanin A	380.39	6	2	1.46	0	Yes
234	Laburnetin	338.35	5	3	1.37	0	Yes
240	Indicanine E	380.39	6	1	1.53	0	Yes
241	Parvisoflavone B	352.34	6	3	1.09	0	Yes
244	Auriculatin	420.45	6	3	2.09	0	Yes
249	4′,7-Dihydroxy-2″,2″- dimethylpyrano [5″,6″:5,6] isoflavone	336.34	5	2	1.64	0	Yes
252	Isoderrone	336.34	5	2	1.64	0	Yes
253	Isochandalon	404.46	5	2	2.64	0	Yes
260	Erymildbraedin B	418.44	6	2	1.76	0	Yes
264	Erysenegalensein F	436.45	7	3	1.37	0	Yes
267	Erysenegalensein L	436.45	7	4	1.29	0	Yes
271	5,7,4′-Trihydroxy-6-(2″-hydroxy3″-methylbut-3″enyl) isoflavone	370.35	7	5	0.29	0	Yes
287	4′-Hydroxyisoflavone-7-O-α-L-rhamnosyl/ (1 →6)-β-ᴅ-glucopyranoside	430.4	9	4	-0.89	0	Yes
436	5,4'-Di-O-methylalpinumisofavone (Dimethylalpinumisoflavone)	378.42	5	0	2.29	0	Yes
440	Erydroogmansin B	420.45	6	3	2.09	0	Yes
444	Erythraddison A	380.39	6	3	1.26	0	Yes
448	Erythrinin G	354.35	6	3	0.9	0	Yes
451	Erythrivarone B	406.47	5	1	2.78	0	Yes
453	Eryvarin G	420.45	6	2	2.36	0	Yes
454	Eryvarin X	406.47	5	3	2.64	0	Yes
458	3’-isoprenylgenistein	338.35	5	3	1.64	0	Yes
464	Ulexone A	388.46	4	1	3.2	0	Yes
289	Demethylmedicarpin	256.25	4	2	1.62	0	Yes
290	Sophorapterocarpan A (Homoedudiol)	324.27	4	2	2.73	0	Yes
293	Erythrabyssin II	392.49	4	2	3.73	0	Yes

Compound No	Compound	Molecular weight	Hydrogen bond acceptor	Hydrogen bond donor	Mlogp	Violation	Druglikeness
299	Erythribyssin B	284.26	5	2	0.96	0	Yes
300	Erythribyssin C	368.42	5	1	2.61	0	Yes
301	Medicarpin	270.28	4	1	1.87	0	Yes
308	Erybraedin B	390.47	4	1	3.73	0	Yes
310	Erybraedin D	390.47	4	1	3.73	0	Yes
312	Erybraedin F	352.38	5	1	2.39	0	Yes
314	Erylysin A	406.47	5	1	2.97	0	Yes
317	Shinpterocarpin	322.35	4	1	2.73	0	Yes
319	Orientanol C	390.47	4	1	3.73	0	Yes
320	Neorautenol	322.35	4	1	2.73	0	Yes
321	Isoneorautenol	322.35	4	1	2.73	0	Yes
322	8-Methoxyneorautenol	352.38	5	1	2.39	0	Yes
323	Phaseollin	322.35	4	1	2.73	0	Yes
324	Folitenol	390.47	4	1	3.73	0	Yes
325	Erythribyssin L	408.49	5	2	2.97	0	Yes
328	Erysubin C	298.29	5	1	1.2	0	Yes
329	Erysubin D	406.478	5	2	2	0	Yes
468	3,9-Dihydroxypterocarp-6a-ene	254.24	4	2	1.46	0	Yes
470	Erythrabissin II	392.49	4	2	3.73	0	Yes
471	Gangetinin	418.48	5	0	3.37	0	Yes
333	Demethylerystagallin A	408.49	5	3	2.9	0	Yes
341	Erysubin E	406.47	5	2	2.9	0	Yes
345	Erycristagallin	390.47	4	2	3.58	0	Yes
352	Coumasterol	268.22	5	2	1.76	0	Yes
354	Sigmoidin K	404.46	5	2	3.88	0	Yes
363	Vignafuran	270.28	4	2	1.71	0	Yes
368	Glyinflanin H	308.33	4	2	2.35	0	Yes
372	2′-O-Demethylbidwillol B	310.34	4	3	2.35	0	Yes

Table S3. Absorption evaluation results of 128 molecular docking hits.

Compound No	Compound	Water solubility	Caco2 permeability	Intestinal absorption (human)	P-glycoprotein substrate	P-glycoprotein I inhibitor	P-glycoprotein II inhibitor
2	Luteolin	-3.139	0.670	84.159	Yes	No	No
3	Vogelin C	-4.151	0.984	84.388	Yes	No	Yes
4	Vogelin J	-3.577	1.101	94.427	Yes	No	Yes
5	Carpachromene	-4.118	1.203	98.392	No	Yes	Yes
379	Abyssinoside A	-2.998	0.153	41.795	Yes	No	No
380	Abyssinoside B	-2.992	-0.035	38.665	Yes	No	No
383	Apigenin	-2.989	0.917	92.502	Yes	No	No
387	Vogeol	-4.031	0.944	92.555	Yes	No	Yes
19	3,7,4′-Trihydroxyflavone	-2.900	1.028	91.733	Yes	No	No
22	Liquiritigenin	-3.655	0.945	91.329	Yes	Np	No
25	Erythribyssin K	-3.551	0.905	93.128	Yes	No	No
26	Liquiritigenin-5′-O-methyl ether	-3.503	0.901	92.701	Yes	No	No
27	7,3′,4′-Trihydroxyflavanone	-3.051	1.004	91.209	Yes	No	No
28	5′-(2-Hydroxy-3-methylbut-3-enyl) abyssinone II	-4.647	0.929	91.765	Yes	Yes	Yes
30	Abyssinone I	-4.497	1.296	94.518	No	No	No
32	Abyssinone III	-6.081	1.724	93.715	Yes	Yes	Yes
35	Erylatissin C	-4.300	0.969	91.228	Yes	Yes	Yes
37	7-Hydroxy-4′-methoxy-3′-(3- methylbut-2-enyl) flavanone	-5.090	1.551	92.758	No	Yes	Yes
41	Erythribyssin I	-3.977	1.118	92.807	Yes	No	No
43	Naringenin	-3.207	0.976	90.044	Yes	No	No
45	Eriodictyol	-3.411	0.759	75.466	Yes	No	No
55	Burttinone	-4.886	0.551	76.712	Yes	Yes	Yes
57	Erycaffra F	-4.783	0.540	68.915	Yes	Yes	Yes
68	2(S)-5,5′,7-Trihydroxy- [2″(5″- hydroxy)-methylpyrano]- (5″,6″:3′,4′) flavanone	-4.210	1.192	92.597	Yes	Yes	No

Compound No	Compound	Water solubility	Caco2 permeability	Intestinal absorption (human)	P-glycoprotein substrate	P-glycoprotein I inhibitor	P-glycoprotein II inhibitor
69	2 (S)-5,7-Dihydroxy-3′-methoxy- [2″(5″-hydroxy)-methylpyrano]- (5″,6″:3′,4′) flavanone	-4.344	1.194	93.355	Yes	Yes	No
79	Fuscaflavanones B	-4.982	1.193	92.025	Yes	Yes	No
80	Abyssinin I	-4.084	1.045	92.847	Yes	Yes	No
84	Abyssinoflavanone V	-3.892	1.110	93.233	Yes	Yes	No
85	Abyssinoflavanone VI	-4.537	0.996	93.811	Yes	Yes	No
90	2(S)-5,7-Dihydroxy- [2″,2″-(3″,4″-dihydroxy)-dimethylpyrano]-(5″,6″:3′,4′) flavanone	-3.209	0.629	70.287	Yes	No	No
92	2(S)-5,5′,7-Dihydroxy-6′-prenyl [2″,2″-(3″,4″-dihydroxy)-dimethylpyrano]-(5″,6″:3′,4′) flavanone	-3.863	0.491	65.684	Yes	Yes	No
391	2S)-5,7-Dihydroxy-3′-prenyl-2″ξ-(4″-hydroxyisopropyl)dihydrofurano[1″,3″:4′,5′] flavanone	-4.113	1.695	82.428	Yes	Yes	No
394	Erylatissin D	-3.851	1.444	89.664	Yes	No	No
396	Erylatissin G	-3.694	1.600	90.496	Yes	No	No
398	2S-3’-(2-Hydroxy-3-methylbut-3-enyl)abyssinone II	-4.094	0.877	91.951	Yes	Yes	Yes
401	4'-Hydroxy-6,3',5'-triprenylisoflavonone	-4.979	0.940	89.174	Yes	Yes	Yes
402	Mildbone	-3.705	1.110	94.945	Yes	No	No
407	Sigmoidin L	-5.489	1.083	91.300	Yes	Yes	Yes
115	Isoliquiritigenin	-3.186	0.930	89.856	Yes	No	No
119	Butein	-2.943	0.339	79.651	Yes	No	No
122	Abyssinone A	-4.075	0.987	92.351	Yes	Yes	No
123	Abyssinone B	-3.912	1.045	91.453	Yes	No	No
124	Abyssinone C	-3.722	0.989	72.650	Yes	No	No
126	2,4,4'-Trihydroxychalcone	-3.237	1.049	72.789	Yes	No	No
416	Mildbone	-3.609	0.952	92.274	Yes	No	No
130	Eryzerin D	-4.787	1.447	94.445	Yes	Yes	Yes
131	Eryzerin C	-3.754	1.281	93.985	Yes	Yes	No
132	Eryvarin T	-3.578	1.076	93.771	Yes	No	No

Compound No	Compound	Water solubility	Caco2 permeability	Intestinal absorption (human)	P-glycoprotein substrate	P-glycoprotein I inhibitor	P-glycoprotein II inhibitor
133	Erythribidin A	-3.683	1.293	93.404	Yes	Yes	No
134	Phaseollinisoflavan	-3.859	1.266	93.154	Yes	No	No
136	Erylivingstone J	-4.384	1.302	94.305	No	Yes	No
137	Erylivingstone K	-3.786	1.253	94.031	Yes	No	No
418	7,4'-Dihydroxy-2',5'-dimethoxyisoflavan	-3.791	1.001	92.747	Yes	No	No
148	Eriotrichin B (Bidwillon A)	-4.100	1.078	93.843	Yes	Yes	No
154	5,2',4'-Trihydroxy-6-prenyl-2‴,2‴dimethyldihydropyrano[5‴,6‴] isoflavanone	-4.217	1.117	95.822	Yes	Yes	Yes
155	5-Deoxyglasperin F	-3.705	1.198	92.738	Yes	Yes	No
156	5-Deoxylicoisoflavanones	-3.970	1.048	94.182	Yes	Yes	No
159	Sigmoidin J	-4.347	1.15	93.410	Yes	Yes	No
160	Bidwillon B	-4.580	1.157	95.253	Yes	Yes	Yes
168	Eryzerin B	-4.378	1.074	95.494	Yes	Yes	Yes
172	2,3-Dihydro-2′-hydroxyosajin	-4.548	0.939	87.038	Yes	Yes	No
174	Erythraddison IV	-3.863	1.104	81.598	Yes	No	No
179	5,3′-Dihydroxy-2″,2″-dimethylpyrano-[5,6:6,7]-2‴,2‴-dimethylpyrano [5,6:5,4] isoflavanone	-5.635	0.636	94.293	Yes	Yes	Yes
181	5,4-Dihydroxy-2-methoxy-8-(3,3- dimethylallyl)-2,2-dimethylpyrano [5,6:6,7] isoflavanone	-4.994	1.129	91.775	Yes	Yes	Yes
183	Licoisoflavanones	-3.951	1.095	81.580	Yes	No	No
422	5,3’-Dihydroxy-5’-(3-hydroxy-3-methyl-1-butenyl)-4’-methoxy-2’’,2’’- dimethylpyrano[5,6:6,7] isoflavanone	-4.549	0.812	87.123	Yes	Yes	Yes
Group VIII	Isoflavones						
190	Daidzein	-3.865	0.805	92.241	Yes	No	No
192	Neobavaisoflavone	-3.806	0.686	93.839	Yes	No	Yes
195	Erythraddison II	-4.002	0.967	92.530	Yes	Yes	Yes
196	2′,7-Dihydroxy-4′-methoxy-5′-(3- methylbut-2-enyl) isoflavone	-4.323	1.044	92.811	Yes	Yes	Yes

Compound No	Compound	Water solubility	Caco2 permeability	Intestinal absorption (human)	P-glycoprotein substrate	P-glycoprotein I inhibitor	P-glycoprotein II inhibitor
197	Calycosin	-3.206	1.062	93.788	Yes	No	No
200	Erylatissin B	-3.953	0.782	93.828	Yes	No	Yes
201	Corylin	-4.731	1.339	96.038	Yes	No	Yes
202	Bidwillon C	-3.782	1.333	96.123	No	No	Yes
209	Isowighteone (3′-Isoprenylgenistein)	-3.443	0.988	92.962	Yes	No	Yes
233	5,4'-Dimethoxy-3'-prenylbiochanin A	-4.569	1.053	95.597	Yes	Yes	Yes
234	Laburnetin	-3.701	0.714	93.855	Yes	No	Yes
240	Indicanine E	-4.388	1.156	94.436	No	Yes	Yes
241	Parvisoflavone B	-4.122	1.206	97.958	Yes	No	Yes
244	Auriculatin	-4.077	0.635	92.655	Yes	No	Yes
249	4′,7-Dihydroxy-2′′,2′′- dimethylpyrano [5′′,6′′:5,6] isoflavone	-3.662	1.12	95.280	Yes	No	Yes
252	Isoderrone	-3.824	1.153	94.650	Yes	No	Yes
253	Isochandalon	-4.513	1.033	94.943	Yes	Yes	Yes
260	Erymildbraedin B	-4.585	0.785	98.815	Yes	Yes	Yes
264	Erysenegalensein F	-4.586	0.992	96.808	Yes	No	Yes
267	Erysenegalensein L	-4.034	1.117	87.345	Yes	No	Yes
271	5,7,4′-Trihydroxy-6-(2′′-hydroxy3′′-methylbut-3′′enyl) isoflavone	-3.031	0.67	70.661	Yes	No	Yes
287	4′-Hydroxyisoflavone-7-O-α-L-rhamnosyl/ (1 →6)-β-ᴅ-glucopyranoside	-3.918	0.335	68.972	Yes	No	Yes
436	5,4'-Di-O-methylalpinumisofavone (Dimethylalpinumisoflavone)	-4.435	1.079	99.638	No	Yes	Yes
440	Erydroogmansin B	-3.813	0.539	89.570	Yes	No	Yes
444	Erythraddison A	-4.155	1.402	96.851	Yes	No	Yes
448	Erythrinin G	-3.911	0.893	97.250	Yes	No	No
451	Erythrivarone B	-4.389	1.042	96.118	Yes	Yes	Yes
453	Eryvarin G	-4.381	1.049	93.222	Yes	Yes	Yes
454	Eryvarin X	-4.349	0.863	93.173	Yes	Yes	Yes

Compound No	Compound	Water solubility	Caco2 permeability	Intestinal absorption (human)	P-glycoprotein substrate	P-glycoprotein I inhibitor	P-glycoprotein II inhibitor
458	3’-isoprenylgenistein	-3.753	0.965	92.349	Yes	No	Yes
464	Ulexone A	-4.571	1.280	96.705	Yes	Yes	Yes
289	Demethylmedicarpin	-3.297	1.204	94.874	Yes	No	No
290	Sophorapterocarpan A (Homoedudiol)	-4.190	1.364	94.279	Yes	Yes	No
293	Erythrabyssin II	-4.766	1.723	93.219	Yes	Yes	Yes
299	Erythribyssin B	-3.443	1.197	95.122	Yes	No	No
300	Erythribyssin C	-4.903	1.448	97.443	No	Yes	No
301	Medicarpin	-3.653	1.294	97.466	No	No	No
308	Erybraedin B	-4.823	1.272	94.832	Yes	Yes	Yes
310	Erybraedin D	-4.754	1.271	95.664	Yes	Yes	Yes
312	Erybraedin F	-4.281	1.703	96.831	No	Yes	Yes
314	Erylysin A	-4.376	1.27	96.678	No	Yes	Yes
317	Shinpterocarpin	-3.952	1.345	96.175	No	No	No
319	Orientanol C	-4.512	1.507	95.744	No	Yes	Yes
320	Neorautenol	-3.846	1.342	96.827	No	No	No
321	Isoneorautenol	-3.999	1.345	96.726	No	No	No
322	8-Methoxyneorautenol	-4.167	1.706	97.483	No	Yes	No
323	Phaseollin	-4.000	1.351	95.894	No	No	No
324	Folitenol	-5.141	1.303	95.316	No	Yes	Yes
325	Erythribyssin L	-4.516	1.423	94.992	Yes	Yes	Yes
328	Erysubin C	-3.845	1.211	98.104	No	No	No
329	Erysubin D	-4.161	1.262	96.402	Yes	Yes	No
468	3,9-Dihydroxypterocarp-6a-ene	-4.167	0.860	90.046	Yes	No	No
470	Erythrabissin II	-4.668	1.362	93.017	Yes	Yes	Yes
471	Gangetinin	-4.587	1.380	96.980	No	Yes	No
333	Demethylerystagallin A	-4.279	0.985	93.660	Yes	Yes	Yes
341	Erysubin E	-4.558	1.229	94.294	Yes	Yes	Yes
345	Erycristagallin	-4.707	0.702	92.110	Yes	Yes	Yes

Compound No	Compound	Water solubility	Caco2 permeability	Intestinal absorption (human)	P-glycoprotein substrate	P-glycoprotein I inhibitor	P-glycoprotein II inhibitor
352	Coumasterol	-4.059	0.921	91.945	Yes	No	No
354	Sigmoidin K	-4.278	1.001	92.708	Yes	Yes	Yes
363	Vignafuran	-3.221	0.956	93.187	Yes	No	No
368	Glyinflanin H	-3.550	1.015	93.798	Yes	No	Yes
372	2′-O-Demethylbidwillol B	-3.658	0.861	92.364	Yes	No	No

Table S4. Distribution evaluation results of 128 molecular docking hits.

Compound No	Compound	VDss (human)	Fraction unbound (human)	BBB permeability	CNS permeability
2	Luteolin	-0.007	0.082	-1.152	-2.455
3	Vogelin C	-0.297	0.117	-1.234	-2.189
4	Vogelin J	0.426	0.156	-0.098	-1.923
5	Carpachromene	0.344	0.214	0.302	-1.789
379	Abyssinoside A	0.179	0.209	-1.649	-4.743
380	Abyssinoside B	0.25	0.223	-1.669	-4.767
383	Apigenin	-0.177	0.209	-0.957	-2.176
387	Vogeol	0.139	0.195	-0.08	-1.757
19	3,7,4′-Trihydroxyflavone	0.139	0.121	-0.893	-2.138
22	Liquiritigenin	-0.13	0.299	0.029	-2.033
25	Erythribyssin K	-0.206	0.274	-0.086	-2.287
26	Liquiritigenin-5′-O-methyl ether	-0.064	0.243	-0.081	-2.238
27	7,3′,4′-Trihydroxyflavanone	-0.08	0.101	-0.894	-2.297
28	5′-(2-Hydroxy-3-methylbut-3-enyl) abyssinone II	0.321	0.094	-0.944	-2.148
30	Abyssinone I	0.179	0.19	0.15	-1.685
32	Abyssinone III	0.386	0.103	-0.146	-1.531
35	Erylatissin C	-0.079	0.19	0.076	-2.018
37	7-Hydroxy-4′-methoxy-3′-(3- methylbut-2-enyl) flavanone	-0.117	0.139	0.04	-1.879
41	Erythribyssin I	-0.02	0.171	-0.113	-2.042
43	Naringenin	-0.234	0.219	-0.811	-2.258
45	Eriodictyol	0.246	0.102	-1.007	-3.21
55	Burttinone	-0.519	0.087	-1.019	-2.867
57	Erycaffra F	-0.492	0.094	-1.139	3.091
68	2(S)-5,5′,7-Trihydroxy- [2″(5″- hydroxy)-methylpyrano]- (5″,6″:3′,4′) flavanone	-0.124	0.164	-1.18	-3.155
69	2 (S)-5,7-Dihydroxy-3′-methoxy- [2″(5″-hydroxy)-methylpyrano]- (5″,6″:3′,4′) flavanone	-0.091	0.155	-1.209	-3.141

Compound No	Compound	VDss (human)	Fraction unbound (human)	BBB permeability	CNS permeability
79	Fuscaflavanones B	0.187	0.104	-0.072	-2.85
80	Abyssinin I	-0.168	0.161	-0.01	-2.944
84	Abyssinoflavanone V	0.048	0.178	0.165	-1.91
85	Abyssinoflavanone VI	-0.002	0.112	0.149	-2.046
90	2(S)-5,7-Dihydroxy- [2′′,2′′-(3′′,4′′-dihydroxy)-dimethylpyrano]-(5′′,6′′:3′,4′) flavanone	0.161	0.146	-1.031	-3.321
92	2(S)-5,5′,7-Dihydroxy-6′-prenyl [2′′,2′′-(3′′,4′′-dihydroxy)-dimethylpyrano]-(5′′,6′′:3′,4′) flavanone	0.511	0.082	-1.419	-3.346
391	2S)-5,7-Dihydroxy-3′-prenyl-2′′ξ-(4′′-hydroxyisopropyl)dihydrofurano[1′′,3′′:4′,5′] flavanone	0.078	0.172	-0.994	-3.011
394	Erylatissin D	0.33	0.201	-1.053	-3.005
396	Erylatissin G	0.376	0.229	-1.047	-2.935
398	2S-3’-(2-Hydroxy-3-methylbut-3-enyl) abyssinone II	0.487	0.117	-1.007	-2.086
401	4'-Hydroxy-6,3',5'-triprenylisoflavonone	-0.081	0.029	-1.001	-1.7
402	Mildbone	0.157	0.087	-0.188	-1.986
407	Sigmoidin L	-0.056	0.082	-0.39	-2.604
115	Isoliquiritigenin	-0.256	0.149	-0.76	-2.323
119	Butein	-0.027	0.132	-0.987	-2.5
122	Abyssinone A	0.025	0.1	-0.145	-1.881
123	Abyssinone B	-0.025	0.103	-0.781	-2.23
124	Abyssinone C	-0.078	0.086	-0.955	-3.172
126	2,4,4'-Trihydroxychalcone	0.14	0.179	-0.944	-2.48
416	Mildbone	0.27	0.073	-1.01	-2.126
130	Eryzerin D	0.428	0	-0.138	-1.715
131	Eryzerin C	0.197	0.077	0.101	-1.865
132	Eryvarin T	0.088	0.1	-0.126	-2.314
133	Erythribidin A	0.414	0.105	-0.626	-1.802
134	Phaseollinisoflavan	0.416	0.066	-0.19	-1.798
136	Erylivingstone J	0.209	0.037	-0.266	-1.59

Compound No	Compound	VDss (human)	Fraction unbound (human)	BBB permeability	CNS permeability
137	Erylivingstone K	0.507	0.086	-0.192	-1.791
418	7,4'-Dihydroxy-2',5'-dimethoxyisoflavan	0.266	0.176	-0.189	-2.203
148	Eriotrichin B (Bidwillon A)	0.19	0.065	-0.894	-2.136
154	5,2',4'-Trihydroxy-6-prenyl-2′′′,2′′′dimethyldihydropyrano[5′′′,6′′′] isoflavanone	-0.356	0.031	0.061	-2.134
155	5-Deoxyglasperin F	0.426	0.096	-0.04	-1.906
156	5-Deoxylicoisoflavanones	0.301	0.085	-0.019	-1.928
159	Sigmoidin J	0.109	0.038	-0.166	-2.764
160	Bidwillon B	0.171	0.008	-0.164	-1.862
168	Eryzerin B	-0.169	0.107	-0.106	-2.004
172	2,3-Dihydro-2′-hydroxyosajin	0.17	0.095	-0.877	-2.898
174	Erythraddison IV	0.441	0.117	-0.958	-2.956
179	5,3′-Dihydroxy-2′′,2′′-dimethylpyrano-[5,6:6,7]-2′′′,2′′′-dimethylpyrano [5,6:5,4] isoflavanone	0.531	0	-0.477	-1.474
181	5,4-Dihydroxy-2-methoxy-8-(3,3- dimethylallyl)-2,2-dimethylpyrano [5,6:6,7] isoflavanone	0.187	0.005	-0.114	-2.632
183	Licoisoflavanones	0.388	0.116	-1.072	-2.921
422	5,3’-Dihydroxy-5’-(3-hydroxy-3-methyl-1-butenyl)-4’-methoxy-2’’,2’’- dimethylpyrano[5,6:6,7] isoflavanone	0.384	0	-1.045	-2.891
190	Daidzein	-0.229	0.142	-0.195	-1.927
192	Neobavaisoflavone	0.233	0.09	0.03	-1.819
195	Erythraddison II	-0.377	0.042	-1.001	-1.81
196	2′,7-Dihydroxy-4′-methoxy-5′-(3- methylbut-2-enyl) isoflavone	0.044	0.063	-0.125	-2.006
197	Calycosin	0	0.109	-0.145	-2.171
200	Erylatissin B	0.379	0.089	-0.109	-1.798
201	Corylin	0.344	0.104	-0.003	-1.598
202	Bidwillon C	0.303	0.162	-0.128	-1.693
209	Isowighteone (3′-Isoprenylgenistein)	-0.012	0.098	-1.045	-2.062
233	5,4'-Dimethoxy-3'-prenylbiochanin A	0.113	0.097	-0.272	-2.087

Compound No	Compound	VDss (human)	Fraction unbound (human)	BBB permeability	CNS permeability
234	Laburnetin	0.37	0.224	-0.986	-2.281
240	Indicanine E	0.204	0.089	-0.318	-1.963
241	Parvisoflavone B	-0.003	0.022	-1.104	-2.143
244	Auriculatin	0.051	0.054	-1.106	-1.863
249	4′,7-Dihydroxy-2″,2″- dimethylpyrano [5″,6″:5,6] isoflavone	0.265	0.109	-0.187	-1.903
252	Isoderrone	0.052	0.111	-0.271	-1.842
253	Isochandalon	0.022	0.085	0.186	-1.72
260	Erymildbraedin B	0.047	0.16	-0.164	-2.039
264	Erysenegalensein F	0.137	0.062	-1.234	-3.231
267	Erysenegalensein L	0.2	0.113	-1.229	-3.278
271	5,7,4′-Trihydroxy-6-(2″-hydroxy3″-methylbut-3″enyl) isoflavone	0.251	0.173	-1.552	-3.486
287	4′-Hydroxyisoflavone-7-O-α-L-rhamnosyl/ (1 →6)-β-D-glucopyranoside	-0.526	0.106	-1.485	-4.023
436	5,4'-Di-O-methylalpinumisofavone (Dimethylalpinumisoflavone)	0.154	0.127	-0.428	-1.909
440	Erydroogmansin B	0.088	0.018	-1.099	-1.862
444	Erythraddison A	0.434	0.096	-1.026	-2.217
448	Erythrinin G	0.199	0.099	-0.989	-2.233
451	Erythrivarone B	0.425	0.243	-0.323	-1.476
453	Eryvarin G	0.26	0.144	-0.185	-1.822
454	Eryvarin X	-0.256	0.087	-0.999	-1.898
458	3’-isoprenylgenistein	0.002	0.079	-1.037	-2.047
464	Ulexone A	0.554	0.103	-0.294	-1.466
289	Demethylmedicarpin	0.366	0.084	-0.362	-2.029
290	Sophorapterocarpan A (Homoedudiol)	0.344	0.012	-0.483	-1.802
293	Erythrabyssin II	-0.12	0.022	-0.69	-1.615
299	Erythribyssin B	0.328	0.076	-0.106	-2.22

Compound No	Compound	VDss (human)	Fraction unbound (human)	BBB permeability	CNS permeability
300	Erythribyssin C	0.212	0	-0.676	-1.964
301	Medicarpin	0.219	0.087	-0.331	-1.655
308	Erybraedin B	0.152	0.021	-0.242	-1.356
310	Erybraedin D	0.215	0.028	-0.245	-1.376
312	Erybraedin F	0.344	0.042	-0.386	-1.814
314	Erylysin A	0.264	0.009	-0.16	-1.682
317	Shinpterocarpin	0.391	0.041	-0.165	-1.776
319	Orientanol C	0.102	0.028	-0.332	-1.475
320	Neorautenol	0.386	0.046	-0.158	-1.783
321	Isoneorautenol	0.405	0.049	-0.087	-1.676
322	8-Methoxyneorautenol	0.32	0.049	-0.38	-1.812
323	Phaseollin	0.326	0.039	-0.084	-1.656
324	Folitenol	0.367	0	-0.224	-1.587
325	Erythribyssin L	-0.052	0.026	0.092	-1.806
328	Erysubin C	0.08	0.093	-0.6	-2.242
329	Erysubin D	0.221	0.035	-0.014	-2.078
468	3,9-Dihydroxypterocarp-6a-ene	-0.122	0.176	0.249	-1.756
470	Erythrabissin II	-0.07	0.075	-0.331	-1.59
471	Gangetinin	0.054	0	-0.135	-1.676
333	Demethylerystagallin A	0.157	0	-0.691	-1.882
341	Erysubin E	0.332	0	0.172	-1.794
345	Erycristagallin	-0.171	0	-0.13	-1.485
352	Coumasterol	-0.255	0.231	-0.401	-1.856
354	Sigmoidin K	-0.49	0.037	-0.193	-1.553
363	Vignafuran	0.28	0.162	-0.115	-1.947
368	Glyinflanin H	0.504	0.15	-0.179	-1.65
372	2′-O-Demethylbidwillol B	0.118	0.07	-0.977	-1.91

Table S5. Metabolism evaluation results of 128 molecular docking hits.

Compound No	Compound	CYP2D6 substrate	CYP3A4 substrate	CYP1A2 inhibitor	CYP2C19 inhibitor	CYP2C9 inhibitor	CYP2D6 inhibitor	CYP3A4 inhibitor
2	Luteolin	No	No	Yes	No	No	No	Yes
3	Vogelin C	No	No	Yes	Yes	Yes	No	No
4	Vogelin J	No	No	Yes	Yes	Yes	No	Yes
5	Carpachromene	No	Yes	Yes	Yes	Yes	No	Yes
379	Abyssinoside A	No	No	No	No	No	No	No
380	Abyssinoside B	No	No	No	No	No	No	No
383	Apigenin	No	No	Yes	No	No	No	Yes
387	Vogeol	No	No	Yes	Yes	Yes	No	Yes
19	3,7,4′-Trihydroxyflavone	No	No	Yes	No	Yes	No	Yes
22	Liquiritigenin	No	No	Yes	Yes	No	No	Yes
25	Erythribyssin K	No	No	Yes	Yes	Yes	No	No
26	Liquiritigenin-5′-O-methyl ether	No	No	Yes	Yes	Yes	No	No
27	7,3′,4′-Trihydroxyflavanone	No	No	Yes	No	No	No	No
28	5′-(2-Hydroxy-3-methylbut-3-enyl) abyssinone II	No	No	No	Yes	Yes	No	Yes
30	Abyssinone I	No	No	Yes	Yes	Yes	No	Yes
32	Abyssinone III	No	No	No	Yes	Yes	No	Yes
35	Erylatissin C	No	No	No	Yes	Yes	No	Yes
37	7-Hydroxy-4′-methoxy-3′-(3-methylbut-2-enyl) flavanone	No	No	Yes	Yes	Yes	No	Yes
41	Erythribyssin I	No	No	Yes	Yes	No	No	No
43	Naringenin	No	No	Yes	Yes	No	No	Yes
45	Eriodictyol	No	No	No	No	No	No	Yes
55	Burttinone	No	No	No	Yes	Yes	No	Yes
57	Erycaffra F	No	No	No	Yes	Yes	No	Yes
68	2(S)-5,5′,7-Trihydroxy- [2″(5″-hydroxy)-methylpyrano]- (5″,6″:3′,4′) flavanone	No	No	Yes	No	No	No	Yes

Compound No	Compound	CYP2D6 substrate	CYP3A4 substrate	CYP1A2 inhibitor	CYP2C19 inhibitor	CYP2C9 inhibitor	CYP2D6 inhibitor	CYP3A4 inhibitor
69	2 (S)-5,7-Dihydroxy-3′-methoxy-[2″(5″-hydroxy)-methylpyrano]-(5″,6″:3′,4′) flavanone	No	No	No	No	No	No	No
79	Fuscaflavanones B	No	No	No	Yes	Yes	No	Yes
80	Abyssinin I	No	No	No	Yes	Yes	No	No
84	Abyssinoflavanone V	No	No	Yes	Yes	Yes	No	No
85	Abyssinoflavanone VI	No	No	Yes	Yes	Yes	No	Yes
90	2(S)-5,7-Dihydroxy- [2″,2″-(3″,4″-dihydroxy)-dimethylpyrano]-(5″,6″:3′,4′) flavanone	No	No	No	No	No	No	No
92	2(S)-5,5′,7-Dihydroxy-6′-prenyl [2″,2″-(3″,4″-dihydroxy)-dimethylpyrano]-(5″,6″:3′,4′) flavanone	No	No	No	No	No	No	No
391	2S)-5,7-Dihydroxy-3′-prenyl-2″ξ-(4″-hydroxyisopropyl)dihydrofurano[1″,3″: 4′,5′] flavanone	No	No	No	Yes	Yes	No	Yes
394	Erylatissin D	No	No	Yes	Yes	Yes	No	No
396	Erylatissin G	No	No	Yes	Yes	Yes	No	Yes
398	2S-3’-(2-Hydroxy-3-methylbut-3-enyl)abyssinone II	No	No	No	Yes	Yes	No	No
401	4'-Hydroxy-6,3',5'-triprenylisoflavonone	No	No	No	Yes	Yes	No	Yes
402	Mildbone	No	No	Yes	Yes	Yes	No	Yes
407	Sigmoidin L	No	No	No	Yes	Yes	No	Yes
115	Isoliquiritigenin	No	No	Yes	Yes	Yes	No	Yes
119	Butein	No	No	Yes	No	No	No	No
122	Abyssinone A	No	No	Yes	Yes	Yes	No	Yes
123	Abyssinone B	No	No	Yes	Yes	Yes	No	No
124	Abyssinone C	No	No	Yes	No	Yes	No	No
126	2,4,4'-Trihydroxychalcone	No	No	Yes	Yes	Yes	No	No

Compound No	Compound	CYP2D6 substrate	CYP3A4 substrate	CYP1A2 inhibitor	CYP2C19 inhibitor	CYP2C9 inhibitor	CYP2D6 inhibitor	CYP3A4 inhibitor
416	Mildbone	No	No	Yes	Yes	Yes	No	No
130	Eryzerin D	No	Yes	No	Yes	Yes	No	Yes
131	Eryzerin C	No	No	Yes	Yes	Yes	No	Yes
132	Eryvarin T	No	No	Yes	Yes	No	No	Yes
133	Erythribidin A	No	No	Yes	Yes	Yes	No	Yes
134	Phaseollinisoflavan	No	No	Yes	Yes	Yes	No	Yes
136	Erylivingstone J	No	Yes	Yes	Yes	Yes	No	Yes
137	Erylivingstone K	No	No	Yes	Yes	Yes	No	Yes
418	7,4'-Dihydroxy-2',5'-dimethoxyisoflavan	No	No	Yes	Yes	No	No	No
148	Eriotrichin B (Bidwillon A)	No	No	Yes	Yes	Yes	No	Yes
154	5,2',4'-Trihydroxy-6-prenyl-2′′′,2′′′dimethyldihydropyrano[5′′′,6′′′] isoflavanone	No	No	Yes	Yes	Yes	No	Yes
155	5-Deoxyglasperin F	No	No	Yes	Yes	Yes	No	No
156	5-Deoxylicoisoflavanones	No	No	Yes	Yes	Yes	No	No
159	Sigmoidin J	No	Yes	No	Yes	Yes	No	Yes
160	Bidwillon B	No	No	No	Yes	Yes	No	Yes
168	Eryzerin B	No	No	No	Yes	Yes	No	Yes
172	2,3-Dihydro-2′-hydroxyosajin	No	No	No	Yes	Yes	No	No
174	Erythraddison IV	No	No	Yes	Yes	No	No	No
179	5,3′-Dihydroxy-2′′,2′′-dimethylpyrano-[5,6:6,7]-2′′′,2′′′-dimethylpyrano [5,6:5,4] isoflavanone	No	Yes	No	Yes	Yes	No	Yes
181	5,4-Dihydroxy-2-methoxy-8-(3,3-dimethylallyl)-2,2-dimethylpyrano [5,6:6,7] isoflavanone	No	Yes	No	Yes	Yes	No	Yes
183	Licoisoflavanones	No	No	Yes	Yes	No	No	No

Compound No	Compound	CYP2D6 substrate	CYP3A4 substrate	CYP1A2 inhibitor	CYP2C19 inhibitor	CYP2C9 inhibitor	CYP2D6 inhibitor	CYP3A4 inhibitor
422	5,3’-Dihydroxy-5’-(3-hydroxy-3-methyl-1-butenyl)-4’-methoxy-2’’,2’’-dimethylpyrano[5,6:6,7] isoflavanone	No	Yes	No	Yes	Yes	No	Yes
190	Daidzein	No	Yes	Yes	Yes	Yes	Yes	No
192	Neobavaisoflavone	No	Yes	Yes	Yes	Yes	No	Yes
195	Erythraddison II	No	No	Yes	Yes	Yes	No	No
196	2′,7-Dihydroxy-4′-methoxy-5′-(3-methylbut-2-enyl) isoflavone	No	No	Yes	Yes	Yes	No	Yes
197	Calycosin	No	No	Yes	Yes	No	No	Yes
200	Erylatissin B	No	No	Yes	Yes	Yes	No	Yes
201	Corylin	No	Yes	Yes	Yes	Yes	No	Yes
202	Bidwillon C	No	No	Yes	Yes	Yes	No	Yes
209	Isowighteone (3′-Isoprenylgenistein)	No	No	Yes	Yes	Yes	No	No
233	5,4'-Dimethoxy-3'-prenylbiochanin A	No	No	Yes	Yes	Yes	No	No
234	Laburnetin	No	No	Yes	Yes	Yes	No	No
240	Indicanine E	No	Yes	Yes	Yes	Yes	No	Yes
241	Parvisoflavone B	No	No	Yes	Yes	Yes	No	No
244	Auriculatin	No	No	No	Yes	Yes	No	No
249	4′,7-Dihydroxy-2″,2″- dimethylpyrano [5″,6″:5,6] isoflavone	No	No	Yes	Yes	Yes	No	Yes
252	Isoderrone	No	No	Yes	Yes	Yes	No	Yes
253	Isochandalon	No	No	Yes	Yes	Yes	No	Yes
260	Erymildbraedin B	No	No	Yes	Yes	Yes	No	No
264	Erysenegalensein F	No	No	No	Yes	Yes	No	No
267	Erysenegalensein L	No	No	No	Yes	Yes	No	No
271	5,7,4′-Trihydroxy-6-(2″-hydroxy3″-methylbut-3″enyl) isoflavone	No	No	Yes	Yes	No	No	No
287	4′-Hydroxyisoflavone-7-O-α-L-rhamnosyl/ (1 →6)-β-ᴅ-glucopyranoside	No	No	No	No	No	No	No

Compound No	Compound	CYP2D6 substrate	CYP3A4 substrate	CYP1A2 inhibitor	CYP2C19 inhibitor	CYP2C9 inhibitor	CYP2D6 inhibitor	CYP3A4 inhibitor
436	5,4'-Di-O-methylalpinumisofavone (Dimethylalpinumisoflavone)	No	Yes	Yes	Yes	Yes	No	Yes
440	Erydroogmansin B	No	No	No	Yes	Yes	No	No
444	Erythraddison A	No	No	Yes	Yes	Yes	No	No
448	Erythrinin G	No	No	Yes	Yes	Yes	No	No
451	Erythrivarone B	No	Yes	Yes	Yes	Yes	No	No
453	Eryvarin G	No	No	No	Yes	Yes	No	Yes
454	Eryvarin X	No	No	Yes	Yes	Yes	No	No
458	3’-isoprenylgenistein	No	No	Yes	Yes	Yes	No	No
464	Ulexone A	No	Yes	Yes	Yes	Yes	No	Yes
289	Demethylmedicarpin	No	No	Yes	Yes	Yes	No	No
290	Sophorapterocarpan A (Homoedudiol)	No	No	Yes	Yes	Yes	No	Yes
293	Erythrabyssin II	No	Yes	No	Yes	Yes	No	Yes
299	Erythribyssin B	No	No	Yes	Yes	No	No	Yes
300	Erythribyssin C	No	Yes	Yes	Yes	Yes	No	Yes
301	Medicarpin	No	Yes	Yes	Yes	Yes	No	No
308	Erybraedin B	No	Yes	No	Yes	Yes	No	Yes
310	Erybraedin D	No	Yes	No	Yes	Yes	No	Yes
312	Erybraedin F	No	Yes	Yes	Yes	Yes	No	Yes
314	Erylysin A	No	Yes	No	Yes	Yes	No	Yes
317	Shinpterocarpin	No	Yes	Yes	Yes	Yes	No	Yes
319	Orientanol C	No	Yes	Yes	Yes	Yes	No	Yes
320	Neorautenol	No	Yes	Yes	Yes	Yes	No	Yes
321	Isoneorautenol	No	Yes	Yes	Yes	Yes	No	Yes
322	8-Methoxyneorautenol	No	Yes	Yes	Yes	Yes	No	Yes
323	Phaseollin	No	Yes	Yes	Yes	Yes	No	Yes
324	Folitenol	No	Yes	No	Yes	Yes	No	Yes
325	Erythribyssin L	No	Yes	No	Yes	Yes	No	Yes
328	Erysubin C	No	No	Yes	Yes	No	No	No

Compound No	Compound	CYP2D6 substrate	CYP3A4 substrate	CYP1A2 inhibitor	CYP2C19 inhibitor	CYP2C9 inhibitor	CYP2D6 inhibitor	CYP3A4 inhibitor
329	Erysubin D	No	Yes	No	Yes	Yes	No	Yes
468	3,9-Dihydroxypterocarp-6a-ene	No	No	Yes	Yes	Yes	Yes	No
470	Erythrabissin II	No	No	No	Yes	Yes	No	Yes
471	Gangetinin	No	Yes	No	Yes	Yes	No	Yes
333	Demethylerystagallin A	No	No	No	Yes	Yes	No	No
341	Erysubin E	No	Yes	No	Yes	Yes	No	Yes
345	Erycristagallin	No	Yes	Yes	Yes	Yes	No	No
352	Coumasterol	No	No	Yes	Yes	Yes	No	Yes
354	Sigmoidin K	No	Yes	Yes	Yes	Yes	No	Yes
363	Vignafuran	No	No	Yes	Yes	Yes	No	Yes
368	Glyinflanin H	No	No	Yes	Yes	Yes	No	No
372	2′-O-Demethylbidwillol B	No	No	Yes	Yes	Yes	No	Yes

Table S6. Excretion evaluation results of 128 molecular docking hits.

Compound No	Compound	Total Clearance	Renal OCT2 substrate
2	Luteolin	-0.615	No
3	Vogelin C	0.702	No
4	Vogelin J	0.582	No
5	Carpachromene	0.74	No
379	Abyssinoside A	0.212	No
380	Abyssinoside B	0.235	No
383	Apigenin	0.693	No
387	Vogeol	0.399	No
19	3,7,4′-Trihydroxyflavone	0.613	No
22	Liquiritigenin	0.084	No
25	Erythribyssin K	0.101	No
26	Liquiritigenin-5′-O-methyl ether	0.124	No
27	7,3′,4′-Trihydroxyflavanone	-0.012	No
28	5′-(2-Hydroxy-3-methylbut-3-enyl) abyssinone II	0.796	No
30	Abyssinone I	0.103	No
32	Abyssinone III	0.588	No
35	Erylatissin C	0.246	No
37	7-Hydroxy-4′-methoxy-3′-(3- methylbut-2-enyl) flavanone	0.206	No
41	Erythribyssin I	0.555	No
43	Naringenin	0.116	No
45	Eriodictyol	0.067	No
55	Burttinone	0.416	No
57	Erycaffra F	0.335	No
68	2(S)-5,5′,7-Trihydroxy- [2″(5″- hydroxy)-methylpyrano]- (5″,6″:3′,4′) flavanone	0.165	No
69	2 (S)-5,7-Dihydroxy-3′-methoxy- [2″(5″-hydroxy)-methylpyrano]- (5″,6″:3′,4′) flavanone	0.21	No
79	Fuscaflavanones B	0.058	No
80	Abyssinin I	0.122	No
84	Abyssinoflavanone V	0.133	No
85	Abyssinoflavanone VI	0.198	No
90	2(S)-5,7-Dihydroxy- [2″,2″-(3″,4″-dihydroxy)-dimethylpyrano]-(5″,6″:3′,4′) flavanone	0.154	No
92	2(S)-5,5′,7-Dihydroxy-6′-prenyl [2″,2″-(3″,4″-dihydroxy)-dimethylpyrano]-(5″,6″:3′,4′) flavanone	0.132	No
391	2S)-5,7-Dihydroxy-3′-prenyl-2″ξ-(4″-hydroxyisopropyl)dihydrofurano[1″,3″:4′,5′] flavanone	0.476	No
394	Erylatissin D	0.33	No
396	Erylatissin G	0.181	No
398	2S-3’-(2-Hydroxy-3-methylbut-3-enyl)abyssinone II	0.837	No
401	4'-Hydroxy-6,3',5'-triprenylisoflavonone	0.478	No
402	Mildbone	0.305	No
407	Sigmoidin L	0.133	No
115	Isoliquiritigenin	0.204	No
119	Butein	0.07	No
122	Abyssinone A	0.202	No
123	Abyssinone B	0.204	No
124	Abyssinone C	0.151	No

Compound No	Compound	Total Clearance	Renal OCT2 substrate
126	2,4,4'-Trihydroxychalcone	0.684	No
416	Mildbone	-0.001	No
130	Eryzerin D	0.315	No
131	Eryzerin C	0.295	No
132	Eryvarin T	0.389	No
133	Erythribidin A	0.208	No
134	Phaseollinisoflavan	0.17	No
136	Erylivingstone J	0.354	No
137	Erylivingstone K	0.307	No
418	7,4'-Dihydroxy-2',5'-dimethoxyisoflavan	0.284	No
148	Eriotrichin B (Bidwillon A)	0.268	No
154	5,2',4'-Trihydroxy-6-prenyl-2‴,2‴dimethyldihydropyrano[5‴,6‴] isoflavanone	0.288	No
155	5-Deoxyglasperin F	-0.005	No
156	5-Deoxylicoisoflavanones	0.042	No
159	Sigmoidin J	0.293	No
160	Bidwillon B	0.343	No
168	Eryzerin B	0.5	No
172	2,3-Dihydro-2′-hydroxyosajin	0.155	No
174	Erythraddison IV	0.232	No
179	5,3′-Dihydroxy-2″,2″-dimethylpyrano-[5,6:6,7]-2‴,2‴-dimethylpyrano [5,6:5,4] isoflavanone	-0.162	Yes
181	5,4-Dihydroxy-2-methoxy-8-(3,3- dimethylallyl)-2,2-dimethylpyrano [5,6:6,7] isoflavanone	0.245	No
183	Licoisoflavanones	0.106	No
422	5,3’-Dihydroxy-5’-(3-hydroxy-3-methyl-1-butenyl)-4’-methoxy-2’’,2’’- dimethylpyrano[5,6:6,7] isoflavanone	0.038	No
190	Daidzein	0.179	No
192	Neobavaisoflavone	0.269	No
195	Erythraddison II	0.446	No
196	2′,7-Dihydroxy-4′-methoxy-5′-(3- methylbut-2-enyl) isoflavone	0.433	No
197	Calycosin	0.241	No
200	Erylatissin B	0.136	No
201	Corylin	0.189	No
202	Bidwillon C	0.264	No
209	Isowighteone (3′-Isoprenylgenistein)	0.338	No
233	5,4'-Dimethoxy-3'-prenylbiochanin A	0.507	No
234	Laburnetin	0.417	No
240	Indicanine E	0.654	No
241	Parvisoflavone B	0.606	No
244	Auriculatin	0.312	No
249	4′,7-Dihydroxy-2″,2″- dimethylpyrano [5″,6″:5,6] isoflavone	0.189	No
252	Isoderrone	0.257	No
253	Isochandalon	0.374	No
260	Erymildbraedin B	0.192	No
264	Erysenegalensein F	-0.215	No
267	Erysenegalensein L	0.489	No
271	5,7,4′-Trihydroxy-6-(2″-hydroxy3″-methylbut-3″enyl) isoflavone	0.626	No
287	4′-Hydroxyisoflavone-7-O-α-L-rhamnosyl/ (1 →6)-β-D-glucopyranoside	2.607	No

Compound No	Compound	Total Clearance	Renal OCT2 substrate
436	5,4'-Di-O-methylalpinumisofavone (Dimethylalpinumisoflavone)	0.41	No
440	Erydroogmansin B	0.337	No
444	Erythraddison A	0.209	No
448	Erythrinin G	0.318	No
451	Erythrivarone B	-0.04	No
453	Eryvarin G	0.347	No
454	Eryvarin X	0.411	No
458	3’-isoprenylgenistein	0.29	No
464	Ulexone A	0.284	No
289	Demethylmedicarpin	0.261	No
290	Sophorapterocarpan A (Homoedudiol)	0.304	No
293	Erythrabyssin II	0.551	No
299	Erythribyssin B	0.12	No
300	Erythribyssin C	0.371	No
301	Medicarpin	0.322	No
308	Erybraedin B	0.111	No
310	Erybraedin D	0.099	No
312	Erybraedin F	0.306	No
314	Erylysin A	-0.076	No
317	Shinpterocarpin	0.13	No
319	Orientanol C	0.077	No
320	Neorautenol	0.274	No
321	Isoneorautenol	0.283	No
322	8-Methoxyneorautenol	0.283	No
323	Phaseollin	0.138	No
324	Folitenol	0.101	No
325	Erythribyssin L	0.161	No
328	Erysubin C	0.25	No
329	Erysubin D	-0.205	No
468	3,9-Dihydroxypterocarp-6a-ene	0.478	No
470	Erythrabissin II	0.614	No
471	Gangetinin	-0.15	No
333	Demethylerystagallin A	0.369	No
341	Erysubin E	0	No
345	Erycristagallin	0.251	No
352	Coumasterol	0.645	No
354	Sigmoidin K	0.874	No
363	Vignafuran	0.665	No
368	Glyinflanin H	0.538	No
372	2′-O-Demethylbidwillol B	0.465	No

Table S7. Toxicity evaluation results of 128 molecular docking hits.

Compound No	Compound	AMES toxicity	hERG I inhibitor	hERG II inhibitor	Hepatotoxicity
2	Luteolin	No	No	No	No
3	Vogelin C	No	No	Yes	No
4	Vogelin J	No	No	Yes	Yes
5	Carpachromene	No	No	Yes	No
379	Abyssinoside A	Yes	No	Yes	No
380	Abyssinoside B	Yes	No	Yes	No
383	Apigenin	No	No	Yes	No
387	Vogeol	No	No	Yes	Yes
19	3,7,4′-Trihydroxyflavone	No	No	Yes	No
22	Liquiritigenin	Yes	No	No	No
25	Erythribyssin K	Yes	No	No	No
26	Liquiritigenin-5′-O-methyl ether	Yes	No	No	No
27	7,3′,4′-Trihydroxyflavanone	No	No	Yes	No
28	5′-(2-Hydroxy-3-methylbut-3-enyl) abyssinone II	No	No	Yes	No
30	Abyssinone I	No	No	No	No
32	Abyssinone III	No	No	Yes	No
35	Erylatissin C	Yes	No	No	No
37	7-Hydroxy-4′-methoxy-3′-(3- methylbut-2-enyl) flavanone	No	No	Yes	No
41	Erythribyssin I	No	No	No	No
43	Naringenin	No	No	No	No
45	Eriodictyol	Yes	No	No	No
55	Burttinone	No	No	Yes	No
57	Erycaffra F	No	No	No	No
68	2(S)-5,5′,7-Trihydroxy- [2″(5″- hydroxy)-methylpyrano]- (5″,6″:3′,4′) flavanone	Yes	No	No	No
69	2 (S)-5,7-Dihydroxy-3′-methoxy- [2″(5″-hydroxy)-methylpyrano]-(5″,6″:3′,4′) flavanone	Yes	No	No	No
79	Fuscaflavanones B	No	No	No	No
80	Abyssinin I	No	No	No	No
84	Abyssinoflavanone V	Yes	No	No	No
85	Abyssinoflavanone VI	No	No	No	No
90	2(S)-5,7-Dihydroxy- [2″,2″-(3″,4″-dihydroxy)-dimethylpyrano]-(5″,6″:3′,4′) flavanone	Yes	No	No	No
92	2(S)-5,5′,7-Dihydroxy-6′-prenyl [2″,2″-(3″,4″-dihydroxy)-dimethylpyrano]-(5″,6″:3′,4′) flavanone	Yes	No	No	No
391	2S)-5,7-Dihydroxy-3′-prenyl-2″ξ-(4″-hydroxyisopropyl)dihydrofurano[1″,3″:4′,5′] flavanone	Yes	No	No	No
394	Erylatissin D	Yes	No	No	No

Compound No	Compound	AMES toxicity	hERG I inhibitor	hERG II inhibitor	Hepatotoxicity
396	Erylatissin G	Yes	No	No	No
398	2S-3’-(2-Hydroxy-3-methylbut-3-enyl) abyssinone II	No	No	Yes	No
401	4'-Hydroxy-6,3',5'-triprenylisoflavonone	No	No	Yes	No
402	Mildbone	No	No	No	No
407	Sigmoidin L	No	No	No	No
115	Isoliquiritigenin	Yes	No	No	No
119	Butein	No	No	No	No
122	Abyssinone A	No	No	Yes	No
123	Abyssinone B	No	No	No	No
124	Abyssinone C	No	No	No	Yes
126	2,4,4'-Trihydroxychalcone	Yes	No	No	No
416	Mildbone	No	No	Yes	No
130	Eryzerin D	No	No	Yes	No
131	Eryzerin C	Yes	No	Yes	No
132	Eryvarin T	Yes	No	Yes	No
133	Erythribidin A	Yes	No	Yes	No
134	Phaseollinisoflavan	Yes	No	Yes	No
136	Erylivingstone J	Yes	No	Yes	No
137	Erylivingstone K	Yes	No	Yes	No
418	7,4'-Dihydroxy-2',5'-dimethoxyisoflavan	Yes	No	No	No
148	Eriotrichin B (Bidwillon A)	No	No	No	No
154	5,2',4'-Trihydroxy-6-prenyl-2′′′,2′′′dimethyldihydropyrano [5′′′,6′′′] isoflavanone	No	No	No	No
155	5-Deoxyglasperin F	Yes	No	No	No
156	5-Deoxylicoisoflavanones	Yes	No	No	No
159	Sigmoidin J	No	No	No	No
160	Bidwillon B	No	No	Yes	No
168	Eryzerin B	No	No	Yes	No
172	2,3-Dihydro-2′-hydroxyosajin	No	No	No	No
174	Erythraddison IV	Yes	No	No	No
179	5,3′-Dihydroxy-2′′,2′′-dimethylpyrano-[5,6:6,7]-2′′′,2′′′-dimethylpyrano [5,6:5,4] isoflavanone	No	No	No	No
181	5,4-Dihydroxy-2-methoxy-8-(3,3- dimethylallyl)-2,2-dimethylpyrano [5,6:6,7] isoflavanone	No	No	No	No
183	Licoisoflavanones	Yes	No	No	No
422	5,3’-Dihydroxy-5’-(3-hydroxy-3-methyl-1-butenyl)-4’-methoxy-2’’,2’’-dimethylpyrano [5,6:6,7] isoflavanone	No	No	No	No
190	Daidzein	No	No	No	No
192	Neobavaisoflavone	No	No	Yes	Yes
195	Erythraddison II	No	No	Yes	No

Compound No	Compound	AMES toxicity	hERG I inhibitor	hERG II inhibitor	Hepatotoxicity
196	2′,7-Dihydroxy-4′-methoxy-5′-(3- methylbut-2-enyl) isoflavone	No	No	Yes	No
197	Calycosin	No	No	Yes	No
200	Erylatissin B	No	No	Yes	Yes
201	Corylin	No	No	Yes	No
202	Bidwillon C	No	No	Yes	No
209	Isowighteone (3′-Isoprenylgenistein)	No	No	Yes	No
233	5,4'-Dimethoxy-3'-prenylbiochanin A	No	No	Yes	No
234	Laburnetin	No	No	Yes	Yes
240	Indicanine E	No	No	Yes	No
241	Parvisoflavone B	No	No	Yes	No
244	Auriculatin	No	No	Yes	No
249	4′,7-Dihydroxy-2′′,2′′- dimethylpyrano [5′′,6′′:5,6] isoflavone	Yes	No	Yes	Yes
252	Isoderrone	No	No	Yes	Yes
253	Isochandalon	No	No	Yes	Yes
260	Erymildbraedin B	No	No	Yes	No
264	Erysenegalensein F	No	No	Yes	No
267	Erysenegalensein L	No	No	Yes	No
271	5,7,4′-Trihydroxy-6-(2′′-hydroxy3′′-methylbut-3′′enyl) isoflavone	No	No	Yes	No
287	4′-Hydroxyisoflavone-7-O-α-L-rhamnosyl/ (1 →6)-β-ᴅ-glucopyranoside	No	No	Yes	Yes
436	5,4'-Di-O-methylalpinumisofavone (Dimethylalpinumisoflavone)	No	No	Yes	Yes
440	Erydroogmansin B	No	No	Yes	No
444	Erythraddison A	No	No	Yes	Yes
448	Erythrinin G	No	No	Yes	No
451	Erythrivarone B	No	No	Yes	No
453	Eryvarin G	No	No	Yes	No
454	Eryvarin X	No	No	Yes	No
458	3’-isoprenylgenistein	No	No	Yes	No
464	Ulexone A	No	No	Yes	No
289	Demethylmedicarpin	Yes	No	No	No
290	Sophorapterocarpan A (Homoedudiol)	Yes	No	Yes	No
293	Erythrabyssin II	No	No	Yes	No
299	Erythribyssin B	No	No	Yes	No
300	Erythribyssin C	No	No	No	No
301	Medicarpin	Yes	No	Yes	No
308	Erybraedin B	No	No	Yes	No
310	Erybraedin D	No	No	Yes	No
312	Erybraedin F	No	No	No	No
314	Erylysin A	Yes	No	No	No
317	Shinpterocarpin	No	No	No	Yes

Compound No	Compound	AMES toxicity	hERG I inhibitor	hERG II inhibitor	Hepatotoxicity
319	Orientanol C	No	No	Yes	No
320	Neorautenol	No	No	Yes	No
321	Isoneorautenol	No	No	Yes	No
322	8-Methoxyneorautenol	No	No	No	No
323	Phaseollin	No	No	Yes	No
324	Folitenol	No	No	Yes	No
325	Erythribyssin L	No	No	Yes	No
328	Erysubin C	Yes	No	No	No
329	Erysubin D	No	No	No	No
468	3,9-Dihydroxypterocarp-6a-ene	Yes	No	No	No
470	Erythrabissin II	No	No	Yes	No
471	Gangetinin	No	No	No	No
333	Demethylerystagallin A	No	No	Yes	No
341	Erysubin E	No	No	Yes	No
345	Erycristagallin	No	No	Yes	No
352	Coumasterol	Yes	No	Yes	No
354	Sigmoidin K	Yes	No	Yes	No
363	Vignafuran	No	No	Yes	No
368	Glyinflanin H	No	No	Yes	No
372	2′-O-Demethylbidwillol B	No	No	Yes	No

Table S8. Interactions between 119 and nsp12 of RdRp and the RNA template and primer. The prefix P indicates primer chain, while T denotes template chain.

Interaction Category	Interaction Type	Ligand/Atom	Pose				
			1	2	3	4	5
Hydrogen Bond	Conventional Hydrogen Bond	119:H10			P:U18		P:U18
Hydrogen Bond	Conventional Hydrogen Bond	119:H11	P:U18				
Hydrogen Bond	Conventional Hydrogen Bond	119:H9	P:U18				
Hydrogen Bond	Conventional Hydrogen Bond	119:O17	P:A19				
Hydrogen Bond	Conventional Hydrogen Bond	119:O17	P:A19				
Hydrogen Bond	Conventional Hydrogen Bond	119:O17	P:U20				
Hydrogen Bond	Conventional Hydrogen Bond	119:H10	P:U20				
Hydrogen Bond	Conventional Hydrogen Bond	119:H11		P:U20	P:U20		
Hydrogen Bond	Conventional Hydrogen Bond	119:O20				P:U20	P:U20
Hydrogen Bond	Carbon Hydrogen Bond	119:O20				P:U20	P:U20
Hydrogen Bond	Conventional Hydrogen Bond	119:H10	T:U12				
Hydrogen Bond	Conventional Hydrogen Bond	119:H11		T:U12	T:U12		
Hydrogen Bond	Conventional Hydrogen Bond	119:H9		T:U12	T:U12	T:U12	T:U12
Hydrogen Bond	Conventional Hydrogen Bond	119:H9		T:U12	T:U12	T:U12	T:U12
Hydrogen Bond	Conventional Hydrogen Bond	119:O17			T:A13		
Hydrogen Bond	Conventional Hydrogen Bond	119:O17			T:A13		T:A13
Hydrogen Bond	Conventional Hydrogen Bond	119:O19		T:A14			
Hydrogen Bond	Conventional Hydrogen Bond	119:O19		T:A14			
Hydrogen Bond	Conventional Hydrogen Bond	119:O10	Gly590				
Hydrogen Bond	Conventional Hydrogen Bond	119:O17				Thr591	
Hydrogen Bond	Conventional Hydrogen Bond	119:O17				Lys593	
Hydrophobic	Pi-Pi T-shaped	119	T:A13			T:A13	T:A13
Hydrophobic	Pi-Pi T-shaped	119	T:A13	T:A13	T:A13	T:A13	T:A13
Hydrophobic	Pi-Pi T-shaped	119				T:A14	
Hydrophobic	Pi-Alkyl	119	Ile589	Ile589	Ile589	Ile589	Ile589
Hydrophobic	Pi-Alkyl	119	Lys593	Lys593	Lys593	Lys593	Lys593
Hydrophobic	Pi-Alkyl	119	Ala688	Ala688	Ala688	Ala688	Ala688
Hydrophobic	Pi-Alkyl	119	Leu758				
Hydrophobic	Pi-Sigma	119		Leu758	Leu758	Leu758	Leu758

Table S9. Descriptive statistics of RMSD values of RdRp-bound **119** and other reference ligands. The reference ligands are Baecalein, adenosine triphosphaste (ATP), and remdesivir triphosphate (RTP).

Complex	Maximum (Å)	Median (Å)	Mean (Å)	IQR (Å)	MAD (Å)	SD (Å)
119	1.442	0.375	0.390	0.163	0.120	0.131
Baecalein	0.815	0.450	0.436	0.147	0.108	0.367
ATP	0.797	0.451	0.436	0.144	0.106	0.120
RTP	2.841	2.132	2.018	0.550	0.306	0.121

Table S10. Descriptive statistics of RMSD values of RdRp (nsp12) binding butein **119** and other reference ligands. The reference ligands are Baecalein, adenosine triphosphaste (ATP), and remdesivir triphosphate (RTP).

Ligand	Maximum (Å)	Median (Å)	Mean (Å)	IQR (Å)	MAD (Å)	SD (Å)
RdRp-**119**	2.728	2.171	2.146	0.185	0.136	0.166
RdRp-Baecalein	2.609	2.083	2.075	0.252	0.185	0.171
RdRp-ATP	3.234	2.701	2.592	0.253	0.179	0.354
RdRp-RTP	2.525	1.909	1.891	0.192	0.138	0.162
Apo	2.233	1.675	1.589	1.780	0.191	0.139

Table S11. Median values of MMGBSA binding energy and their energy terms of 119, Baecalein, ATP, and RTP to RdRp (nsp12).

Ligand	MMGBSA Binding Energy (kcal mol^{-1})	MMGBSA Energy Terms (kcal mol^{-1})			
		vdW	Electrostatics	EGB	ESurf
**119**	–45.98	–50.97	–18.95	29.21	–4.88
Baecalein	–24.09	–38.17	–10.05	28.50	–4.76
ATP	–33.59	–9.97	–319.28	300.57	–4.66
RTP	–55.91	–7.61	–38.79	–11.56	–4.77

Table S12. Median values of MMGBSA per-residue energy decomposition and their energy terms of **119** binding to RdRp (nsp12).

Residue	MMGBSA Energy Terms (kcal mol⁻¹)				MMGBSA Binding Energy (kcal mol⁻¹)
	Electrostatic	vdW	Non-Polar Solvation	Polar Solvation	
P:A19	-3.14	-3.56	-1.95	2.43	-6.02
T:A13	0.32	-3.72	-1.73	0.27	-4.80
P:U20	-0.30	-2.99	-1.25	0.68	-3.85
Thr591	-0.90	-1.80	-1.01	0.22	-3.59
Ile589	-0.87	-1.32	-0.92	-0.23	-3.34
P:U18	-4.25	-0.62	-0.64	2.64	-2.73
Lys593	2.65	-1.30	-1.06	-2.90	-2.57
T:A14	-0.27	-1.76	-0.62	0.34	-2.34
T:U12	-1.69	-0.61	-0.59	0.74	-2.05
Leu758	-0.31	-0.98	-0.65	-0.03	-1.98
Cys813	-0.20	-1.26	-0.72	0.30	-1.95
Gly590	-0.54	-0.63	-0.26	0.35	-1.13
Gln815	0.19	-0.47	-0.34	-0.50	-1.08
Ala688	-0.05	-0.44	-0.30	-0.20	-0.96
Ser592	0.10	-0.59	-0.20	-0.18	-0.87
Trp598	0.09	-0.50	-0.34	0.05	-0.66
Val588	0.53	-0.44	-0.10	-0.47	-0.51
Thr687	0.08	-0.05	0.00	-0.23	-0.20
Asn691	-0.01	-0.02	0.00	-0.06	-0.09
Mg1254	-0.46	0.00	0.00	0.41	-0.05
Asp761	0.40	-0.04	0.00	-0.40	-0.04
Ser759	0.11	-0.08	0.00	-0.06	-0.04
T:U10	-0.30	-0.01	0.00	0.29	-0.02
Ser682	0.01	-0.01	0.00	-0.02	-0.02
Asp833	-0.45	0.00	0.00	0.45	-0.01
Val557	0.00	0.00	0.00	-0.01	-0.01
Arg836	1.09	-0.01	0.00	-1.09	-0.01
Mg1255	-0.48	0.00	0.00	0.47	-0.01
Cys622	-0.01	0.00	0.00	0.00	0.00
Arg555	0.27	-0.01	0.00	-0.27	0.00
Ile548	-0.02	0.00	0.00	0.02	0.00
Lys676	-0.14	0.00	0.00	0.13	0.00
Thr556	0.01	0.00	0.00	-0.02	0.00
Asp623	0.33	0.00	0.00	-0.33	0.00
Leu544	-0.01	0.00	0.00	0.01	0.00
Lys798	-0.11	0.00	0.00	0.11	0.00
Ala547	0.03	0.00	0.00	-0.03	0.00

Residue	MMGBSA Energy Terms (kcal mol⁻¹)				MMGBSA Binding Energy (kcal mol⁻¹)
	Electrostatic	vdW	Non-Polar Solvation	Polar Solvation	
Ala554	0.01	0.00	0.00	-0.01	0.00
Asp445	-0.09	0.00	0.00	0.09	0.00
Asp452	-0.04	0.00	0.00	0.04	0.00
Asp618	0.21	0.00	0.00	-0.20	0.00
Ser549	-0.01	0.00	0.00	0.01	0.00
Tyr546	-0.03	0.00	0.00	0.03	0.00
Ala443	0.00	0.00	0.00	0.00	0.00
Arg553	0.13	0.00	0.00	-0.13	0.00
Gln444	-0.01	0.00	0.00	0.01	0.00
Lys551	0.09	0.00	0.00	-0.09	0.00
Phe441	0.01	0.00	0.00	-0.01	0.00
Phe442	-0.01	0.00	0.00	0.01	0.00
T:A11	-0.10	-0.13	-0.01	0.23	0.00
T:U9	-0.22	0.00	0.00	0.22	0.00
Val410	0.01	0.00	0.00	-0.01	0.00
Arg624	-0.07	0.00	0.00	0.07	0.00
Lys438	0.24	0.00	0.00	-0.24	0.00
Lys545	0.60	0.00	0.00	-0.59	0.00
Asp760	0.47	-0.02	0.00	-0.44	0.01

Table S13. H-bond occurrence formed between **119** and RdRp, the RNA primer and template. T in the Acceptor Residue column denotes the RNA template, whereas P is the RNA primer.

Acceptor Residue	Acceptor Atom	Donor Residue	Donor Atom	Frames	H-Bond Fraction	% H-Bond Occurrence
T:U12	O2	119	H10	6959	0.6959	69.59
P:A19	N3	119	H4	5969	0.5969	59.69
P:U18	O2	119	H9	4220	0.422	42.2
119	O17	P:A19	HO2'	1005	0.1005	10.05
P:U18	O2'	119	H9	622	0.0622	6.22
119	O10	Thr591	H	299	0.0299	2.99
119	O19	T:U12	HO2'	190	0.019	1.9
119	O10	Gly590	H	182	0.0182	1.82
T:A13	N3	119	H4	137	0.0137	1.37
U3_1238	O2	119	H10	133	0.0133	1.33
119	O18	P:U18	HO2'	52	0.0052	0.52
119	O20	Gln815	HE21	47	0.0047	0.47
P:U18	O2'	119	H11	37	0.0037	0.37
Cys813	O	119	H11	32	0.0032	0.32
P:A19	O4'	119	H9	28	0.0028	0.28
P:A19	O4'	119	H11	17	0.0017	0.17
P:U18	O2	119	H11	12	0.0012	0.12
Gln815	OE1	119	H11	8	0.0008	0.08
Cys813	O	119	H9	6	0.0006	0.06
119	O20	P:U18	HO2'	6	0.0006	0.06
119	O20	Gln815	HE22	5	0.0005	0.05
Gln815	OE1	119	H9	4	0.0004	0.04
Gln815	NE2	119	H11	3	0.0003	0.03
T:U12	O2'	119	H10	2	0.0002	0.02
119	O18	Lys593	HZ2	2	0.0002	0.02
T:A14	N3	119	H9	1	0.0001	0.01
Thr591	O	119	H11	1	0.0001	0.01
119	O18	Lys593	HZ3	1	0.0001	0.01

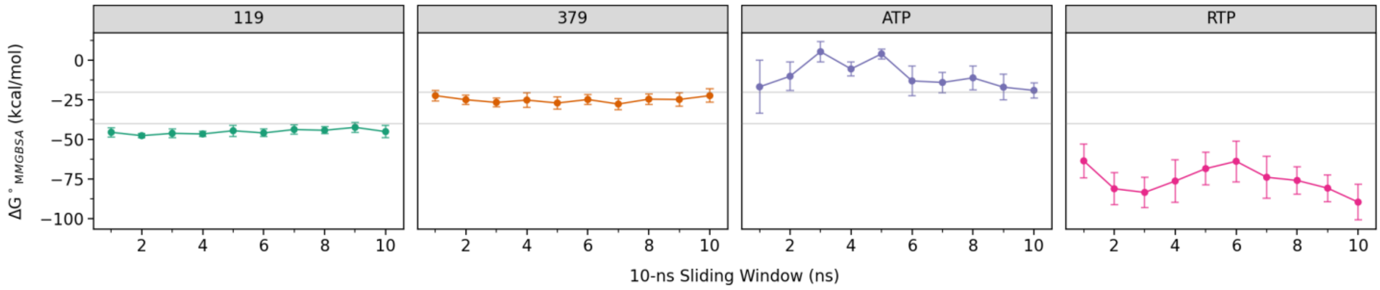

Figure S1. MMGBSA binding energy trajectories of ligands to RdRp over 100 ns. Each data point was computed from a 10-ns sliding window.
